# Mitochondrial-Oriented Injectable Hydrogel Microspheres Maintain Homeostasis of Chondrocyte Metabolism to Promote Subcellular Therapy in Osteoarthritis

**DOI:** 10.34133/research.0306

**Published:** 2024-01-25

**Authors:** Li Chen, Jianye Yang, Zhengwei Cai, Yanran Huang, Pengcheng Xiao, Hong Chen, Xiaoji Luo, Wei Huang, Wenguo Cui, Ning Hu

**Affiliations:** ^1^ Department of Orthopedics, The First Affiliated Hospital of Chongqing Medical University, Orthopedic Laboratory of Chongqing Medical University, Chongqing 400016, China.; ^2^ Department of Orthopaedics, Shanghai Key Laboratory for Prevention and Treatment of Bone and Joint Diseases, Shanghai Institute of Traumatology and Orthopaedics, Ruijin Hospital, Shanghai Jiao Tong University School of Medicine, 197 Ruijin 2nd Road, Shanghai 200025, China.

## Abstract

Subcellular mitochondria serve as sensors for energy metabolism and redox balance, and the dynamic regulation of functional and dysfunctional mitochondria plays a crucial role in determining cells' fate. Selective removal of dysfunctional mitochondria at the subcellular level can provide chondrocytes with energy to prevent degeneration, thereby treating osteoarthritis. Herein, to achieve an ideal subcellular therapy, cartilage affinity peptide (WYRGRL)-decorated liposomes loaded with mitophagy activator (urolithin A) were integrated into hyaluronic acid methacrylate hydrogel microspheres through microfluidic technology, named HM@WY-Lip/UA, that could efficiently target chondrocytes and selectively remove subcellular dysfunctional mitochondria. As a result, this system demonstrated an advantage in mitochondria function restoration, reactive oxygen species scavenging, cell survival rescue, and chondrocyte homeostasis maintenance through increasing mitophagy. In a rat post-traumatic osteoarthritis model, the intra-articular injection of HM@WY-Lip/UA ameliorated cartilage matrix degradation, osteophyte formation, and subchondral bone sclerosis at 8 weeks. Overall, this study indicated that HM@WY-Lip/UA provided a protective effect on cartilage degeneration in an efficacious and clinically relevant manner, and a mitochondrial-oriented strategy has great potential in the subcellular therapy of osteoarthritis.

## Introduction

Osteoarthritis, a prevalent chronic ailment, afflicts over 300 million individuals globally and has emerged as the primary contributor to societal expenses due to the aging of the worldwide populace. Unfortunately, there remains a deficiency in effective interventions that can achieve long-term satisfactory performance, particularly for early-stage osteoarthritis [1]. It is widely acknowledged that chondrocytes constitute the main cellular component in cartilage tissue and mainly govern the maintenance of its homeostatic equilibrium. Recent evidence suggests that mitochondria are crucial organelles that serve as central contributors to catabolic and anabolic processes, apoptosis, and signal transduction [2]. Furthermore, mitochondria-mediated aerobic respiration is critical for the energy metabolism of chondrocytes, even though they are in a relatively hypoxic microenvironment [3]. As osteoarthritis progresses, the mitochondrial respiratory capacity of chondrocytes may diminish due to a decrease in mitochondrial biogenesis. More importantly, chondrocytes in osteoarthritis exhibit a limited ability to eliminate damaged mitochondria, thereby exacerbating the intracellular accumulation of dysfunctional mitochondria. Compounding the issue, dysfunctional mitochondria are a major source of reactive oxygen species (ROS), which leads to promote inflammation responses and extracellular matrix (ECM) degradation, eventually forming a vicious cycle to accelerate cartilage degeneration [4]. However, current therapies for osteoarthritis are generally constrained to restoring chondrocyte function, maintaining phenotypes, and promoting regeneration. These approaches have limitations in addressing fundamental problems with mitochondria [5]. Accordingly, it is promising to develop novel mitochondria-oriented interventions for osteoarthritis that may protect against cartilage degeneration at the subcellular level.

Mitophagy, the primary process for mitochondrial quality control, enables the selective removal of dysfunctional mitochondria, thereby reducing oxidative stress and inflammation levels in chondrocytes [6]. Increasing evidence has demonstrated that the PINK1-Parkin pathway contributes to ubiquitinating the mitochondrial membrane, subsequently binding to LC3B to activate the mitophagy program effectively. This process has been implicated in the protective effects against osteoarthritis development [7]. Despite there being a number of strategies aimed at enhancing mitochondrial function with a focus on stimulating mitochondrial biogenesis or targeting the respiratory chain, research on specific and safe mitophagy inducers for osteoarthritis treatment is currently insufficient [8]. Urolithin A (UA) is generated by the intestinal microbiome through the conversion of ellagitannins and ellagic acid, making it a naturally occurring postbiotic agent. Accumulating evidence has shown that UA has the ability to improve muscle strength and endurance through inducing mitophagy in the preclinical models of muscle dystrophy and aging [9,10]. With enhanced mitochondrial function, UA was also reported to have promising results in conditions such as heart failure, inflammatory bowel disease, and neurodegenerative disorders [11–13]. Furthermore, clinical studies proved that UA is both safe and effective in improving exercise performance and biomarkers of mitophagy [14]. In this context, UA is considered to have the potential to attenuate the pathogenesis of osteoarthritis by enhancing mitophagy in chondrocytes. The key to implementing the restoration of normal mitochondrial metabolism is to ensure that UA functions precisely in the chondrocytes.

Currently, intra-articular injections of drugs are popularly used in osteoarthritis treatment. As previously reported, antioxidants such as curcumin, bilobalide, and resveratrol showed antioxidant effects against chondrocyte apoptosis [15–17]. However, these agents were observed to readily interact with constituents in synovial fluid, thereby decreasing their antioxidant efficiency and suggesting that this approach may not precisely target chondrocytes. Considering that the cartilage ECM primarily consists of type II collagen and proteoglycan, delivering drugs to chondrocytes and achieving a protective effect poses a great challenge [18]. In recent years, intra-articular injections of nanocarriers loaded with effective drugs for the treatment of osteoarthritis have been prevalently developed, proving a promising strategy to achieve targeted therapy. Among these nanomaterials, liposomes, characterized by excellent properties such as high cycling stability, bilayer structure resembling the cellular membrane, and the ability to penetrate natural barriers, have attracted tremendous attention across various fields. Through surface modifications, liposomes may exhibit enhanced targeting ability, which is conducive to increasing local concentration, efficiency, and engineerability [19]. It is worth mentioning that WYRGRL (termed WY), one of the cartilage affinity peptides, was found to significantly improve the chondrocyte targeting efficiency of nanoplatform in vivo [20]. Xue et al. [21] found that WY-functionalized exosomes exhibited a strong chondrocyte-targeting effect, which successfully enhanced the cellular uptake of exosomes, and could exert protective effects on the murine model of osteoarthritis via intra-articular administration. As for these, WY was selected for further modification onto the liposomes to obtain efficient and targeted delivery of UA to chondrocytes.

**Fig. 1.**
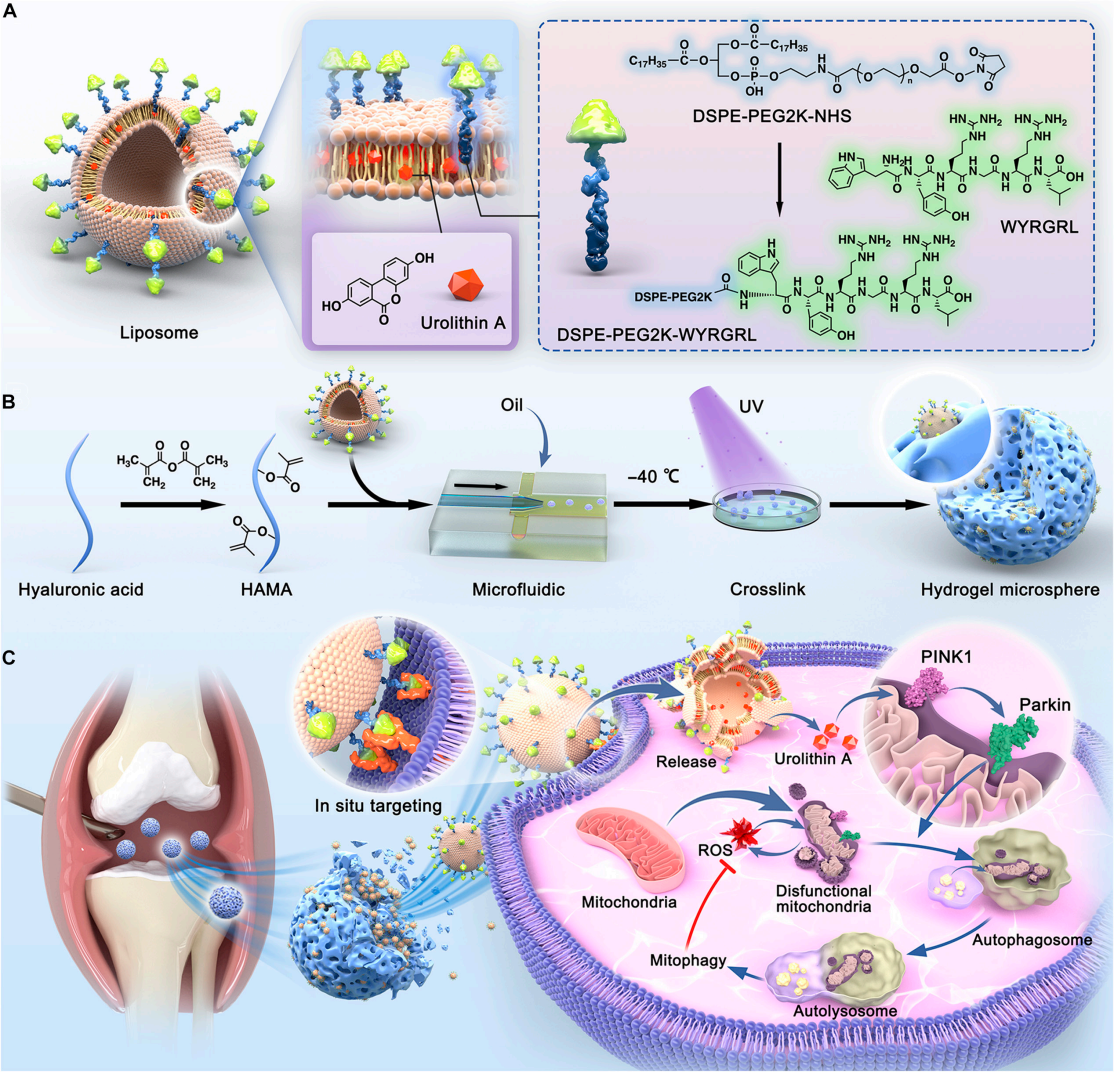
Schematic diagram of preparation and regulation of the mitochondrial dynamic-oriented hydrogel microspheres. (A) The synthesis of WY-decorated liposomes loaded with UA (WY-Lip/UA). (B) The construction of microfluidic hydrogel microspheres (HM@WY-Lip/UA). (C) Illustration of the injection of HM@WY-Lip/UA for treatment of osteoarthritis by activating PINK1-Parkin-mediated mitophagy.

Within the articular cavity, small-sized liposomes are prone to clearance by lymphatics and capillaries, and only a sufficiently large net inflow can ensure an adequate concentration of therapeutic drugs before clearance occurs [22]. However, a substantial increase in the injectable dose should consider the safety and potential side effects of the drug [23]. Therefore, extending the retention time of liposomes within the joint space, while optimizing their role in targeting chondrocytes, is a bottleneck that needs to be addressed. Hydrophilic polymer-based hydrogel microspheres (HMs) are micron-sized spherical structures with outstanding biocompatibility and injectability [24]. They can remain within the joint cavity for a prolonged duration and effectively reduce friction in a manner similar to ball bearings [25]. To achieve an ideal subcellular therapy, WY-decorated liposomes loaded with UA (WY-Lip/UA) were integrated into a hyaluronic acid methacrylate (HAMA) matrix through microfluidic technology and photopolymerization processes, producing mitochondrial-oriented injectable HM@WY-Lip/UA. In this system, HM effectively served as reservoirs for liposomes to continuously expose and successfully target chondrocytes, thereby maintaining cellular homeostasis by inducing mitophagy. Eventually, engineered multifunctional HM@WY-Lip/UA were delivered locally in a rat osteoarthritis model (Fig. 1). The developed mitochondrial-oriented injectable HM@WY-Lip/UA is suitable for the treatment of degenerative diseases such as osteoarthritis.

## Results and Discussion

### Characterization of mitochondrial-oriented injectable HM@WY-lip/UA

As drug carriers, liposomes are appropriate for carrying various drugs, featuring an internal aqueous core suitable for hydrophilic drugs and a bilayer for hydrophobic drugs [26]. Furthermore, liposomes play a role in regulating drug release and reducing drug adverse reactions. To achieve drug targeted delivery, the phospholipid bilayer can serve as an excellent platform for the modification with targeting peptides [27]. In this study, WY peptide was chosen to functionalize liposomes for chondrocyte targeting; thus, DSPE-PEG2K-NHS was initially selected to graft WY in preparation for the synthesis of liposomes. The proton nuclear magnetic resonance (^1^H NMR) spectra of DSPE-PEG2K-WY confirmed that WY was successfully conjugated to DSPE-PEG2K (Fig. S1).

**Fig. 2.**
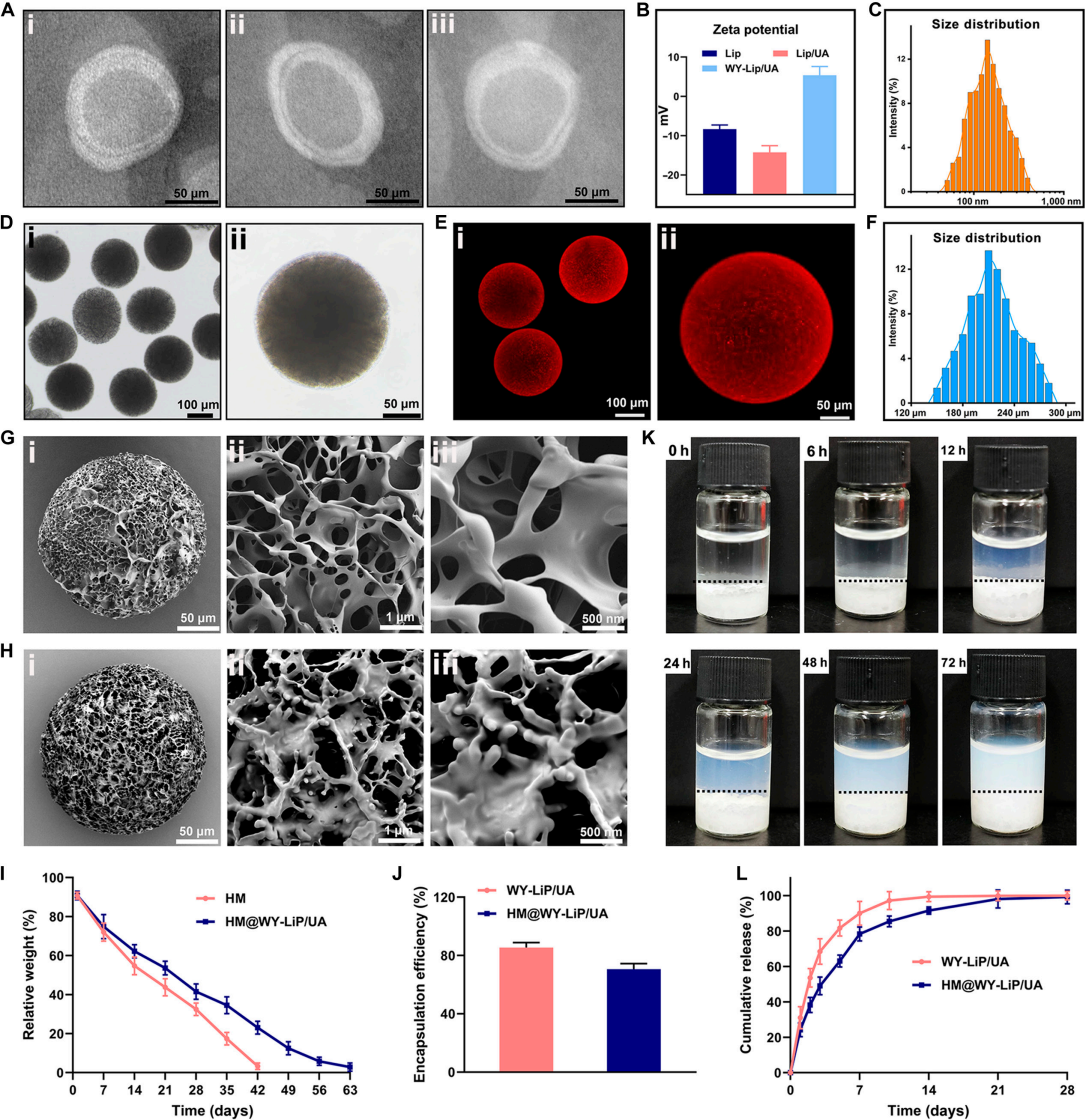
Characterization of liposomes and hydrogel microspheres. (A) TEM observation of (i) Lip, (ii) Lip/UA, and (iii) WY-Lip/UA. (B) Zeta potential of liposomes. (C) Size distribution of WY-Lip/UA. (D) The morphology of HM@WY-Lip/UA under bright-field microscopy: (i) dispersed HM@WY-Lip/UA and (ii) individual HM@WY-Lip/UA. (E) LSCM images of HM@WY-Lip/UA: (i) dispersed HM@WY-Lip/UA and (ii) individual HM@WY-Lip/UA. (F) Size distribution of HM@WY-Lip/UA. (G) SEM observation of pure HM from (i) the overall view and (ii and iii) the local view. (H) SEM observation of HM@WY-Lip/UA from (i) the overall view and (ii and iii) the local view. (I) In vitro degradation of HM@WY-Lip/UA. (J) Encapsulation efficiency of UA in HM and HM@WY-Lip/UA. (K) Liposome release from HM to the solution. (L) Cumulative release of UA from WY-Lip/UA and HM@WY-Lip/UA. *n* = 3 for each group.

In general, the thin-film dispersion technique is suitable for the preparation of liposomes loaded with fat-soluble drugs, and a high encapsulation rate can be achieved [28]. As shown in Fig. 2A, blank liposomes (Lip), liposomes loaded with UA (Lip/UA), and WY-Lip/UA were successfully constructed by the thin-film dispersion method, and these 3 liposomes exhibited a typical bilayer structure as confirmed by transmission electron microscope (TEM) analysis. WY-Lip/UA displayed a positive zeta potential of 5.4 ± 2.2 mV (Fig. 2B), which was more likely to have affinity with the glycosaminoglycan chains carrying a negative charge in cartilage [29]. In addition, the average diameters of Lip and Lip/UA were 102.8 ± 16.4 nm and 109.7 ± 17.2 nm, respectively (Fig. S2). However, WY-Lip/UA displayed a good dispersity with a larger average size of 138.2 ± 27 nm (Fig. 2C). A potential explanation for this inconsistency is that the proportion of lecithin has the capacity to regulate the diameter of liposomes, and the modification of DSPE-PEG2K-WY may introduce a dense conformational cloud to the liposomes. Given that UA needs to enter the interior of chondrocytes to exert its activity, small-sized liposomes (under 200 nm) are advantageous for cartilage penetration and facilitating the intracellular transport of UA [30].

For an ideal mitochondria-oriented intervention, HM should not only be adapted to the joint immune microenvironment but also efficiently load and deliver WY-Lip/UA. Hyaluronic acid (HA), a major ECM component in articular cartilage, has been commonly employed in the treatment of osteoarthritis [31]. HAMA is produced by modifying the methacrylate groups on the molecular chain of HA, providing it with the capability for cross-linking upon exposure to ultraviolet (UV) light. The degree of methylation of HAMA can affect the characterization of HM, such as stiffness, biodegradation, and pore structure [32]. In this study, the ^1^H NMR spectra of HAMA revealed a 64.8% methacrylation, confirming the effective incorporation of methacrylate groups (Fig. S3). Following this, HAMA HMs were fabricated using microfluidic technology, displaying a well-dispersed structure with consistent dimensions and shape as observed under optical microscopy (Fig. 2D). Research indicated that small-sized HMs were suitable for intra-articular injection, yet excessively small diameters were not conducive to diffusion within the joint [33]. Typically, HMs with a diameter between 200 and 300 μm are preferred for intra-articular injection [34]. As a result, HMs with a diameter of 212.9 ± 13.6 μm were harvested in our study through adjusting flow rates of continuous and dispersed phases (Fig. 2F). Laser scanning confocal microscopy (LSCM) further confirmed that DiI-labeled WY-Lip/UA were successfully loaded into HM (Fig. 2E).

Scanning electron microscopy (SEM) confirmed the presence of a porous microstructure in the HM prepared in this study, characterized by a pronounced interconnectivity (Fig. 2G and H). The porous microstructure contributes to the high specific surface area of HM, facilitating high-capacity loading of liposomes [35]. Interestingly, only a few liposomes were found on the outer surface of HM after being loaded. This could be attributed to the majority of liposomes being encapsulated within the HAMA. Moreover, the liposomes remaining on the outer surface may have been cleared in the process of oil removal without protection from the hydrogel network. According to the degradation experiments in vitro, the degradation rate of HM was relatively rapid, with complete degradation observed by 6 weeks. HM@WY-Lip/UA degraded at a similar rate to HM in the first week and then displayed a relatively slower degradation rate until 9 weeks before complete degradation (Fig. 2I). This phenomenon may be attributed to the liposomes released from HM during degradation, which produced a hydrated layer around it and thus forming a physical barrier to hyaluronidase to some extent, thereby slowing down the degradation rate [36].

UA has been found to possess low bioavailability attributed to its poor aqueous solubility and short elimination half-life [37]. By encapsulating UA within liposomes, it is possible to prolong in vivo retention time and increase cellular uptake [38]. Moreover, the stability of drug release from liposomes can be further improved through the combination with HM, which may be beneficial for local drug delivery [39]. According to the data presented in Fig. 2J, the encapsulation efficiency of WY-Lip/UA and HM@WY-Lip/UA was 85.4% ± 5.9% and 70.7% ± 6.4%, respectively. The reduction in encapsulation efficiency following incorporation within HMs may be attributed to drug leakage and partial loss of liposomes during the oil-removal procedure [40]. As shown in Fig. 2K, it was observed that liposomes were released from the microspheres, leading to a change in the appearance of the liquid in the upper part of the microspheres from transparent to milky white after 72 h. This indicated that the porous structure of HM provided a route for liposomes to traverse and release into the external environment of the HM. Besides, the cumulative release profiles of UA from WY-Lip/UA and HM@WY-Lip/UA are shown in Fig. 2L. In both groups, an initial rapid release was observed within the initial 72-h period, subsequently transitioning to a slower release rate. Overall, HM@WY-Lip/UA presented a relatively sustained release compared to WY-Lip/UA, suggesting that HM@WY-Lip/UA can be chosen as the appropriate carrier for local delivery of UA.

### Chondrocyte targeting ability and biocompatibility of HM@WY-lip/UA

**Fig. 3.**
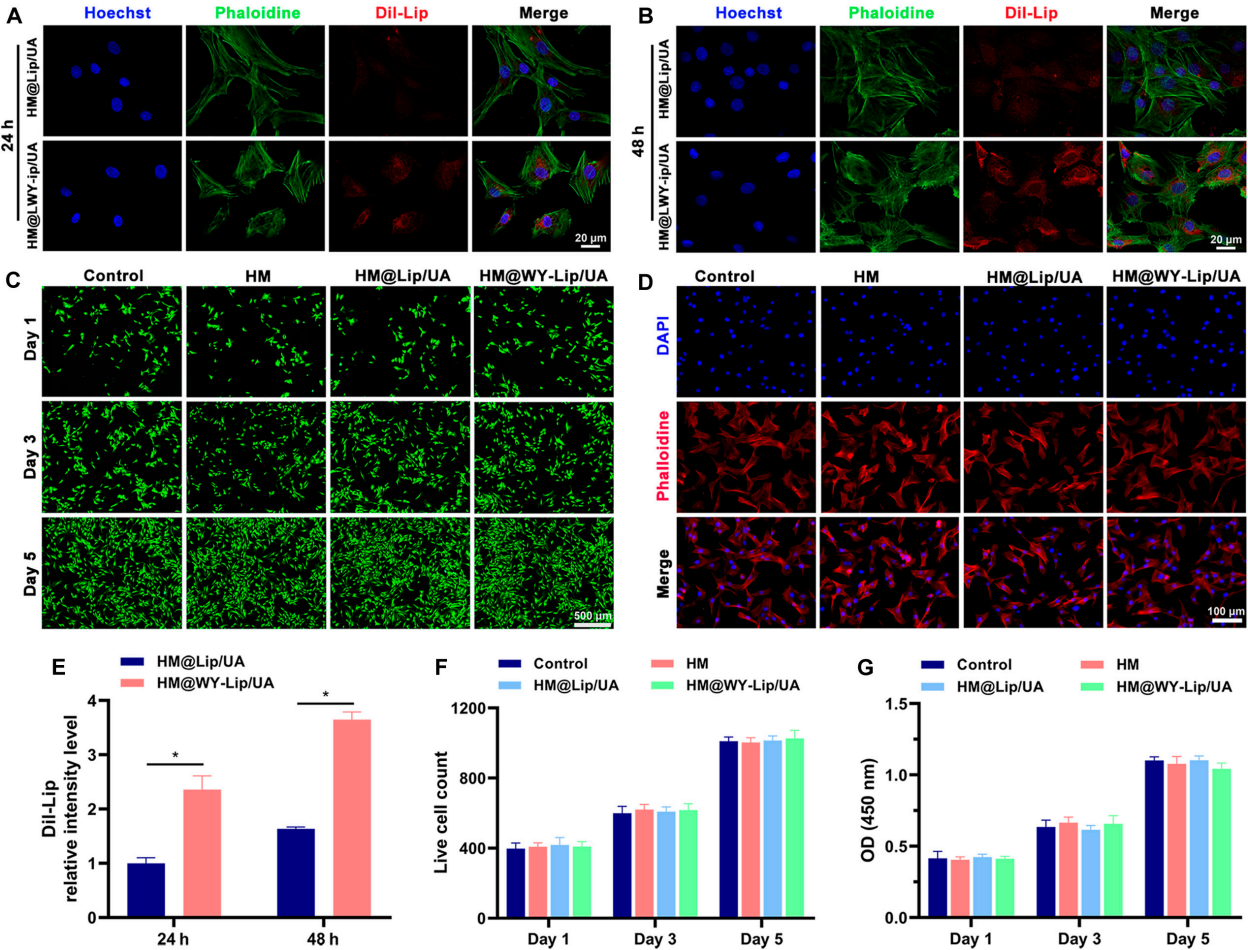
Evaluation of chondrocyte targeting ability and biocompatibility of HM@WY-Lip/UA. (A) Cellular uptake of liposomes from HM@WY-Lip/UA at 24 h. (B) Cellular uptake of liposomes from HM@WY-Lip/UA at 48 h. (C) Live/dead staining of chondrocytes co-culture. (D) The results of cytoskeleton staining. (E) Quantitative analysis of liposomes cellular uptake. (F) Live/dead staining quantitative analysis. (G) CCK-8 assay results of chondrocytes co-culture. *n* = 3 for each group; * indicates statistical significance (*P* < 0.05).

Recently, accumulating evidence has indicated that targeted delivery of biologicals toward chondrocytes appears to be a promising strategy for osteoarthritis treatment, and there has been a surge in interest surrounding a diverse range of peptides that exhibit specific affinity to cartilage components [41]. Based on this, WY was grafted onto liposomes to develop a more efficient delivery of UA to chondrocytes in this study. As seen from Fig. 3A, B, and E, a noticeable increase in the red fluorescence signals of liposomes within the chondrocytes was observed in the HM@WY-Lip/UA group both after 24 and 48 h of co-culture, indicating that the addition of WY indeed improved the chondrocyte targeting ability of this platform. In addition, the influences of the HM@WY-Lip/UA on cell viability were further assessed by live/dead cell staining along with CCK-8 assay. Based on the observations depicted in Fig. 3C, F, and G, there was no discernible disparity in cell count among the various groups, and it is noteworthy that the quantity of viable chondrocytes exhibited a certain degree of augmentation in all groups on day 5. Meanwhile, cytoskeleton staining displayed similar outcomes in terms of well-maintained cellular morphology among chondrocytes in each group (Fig. 3D). Accordingly, HM@WY-Lip/UA demonstrated to have a favorable biocompatibility.

### Restoration of mitochondrial function

**Fig. 4.**
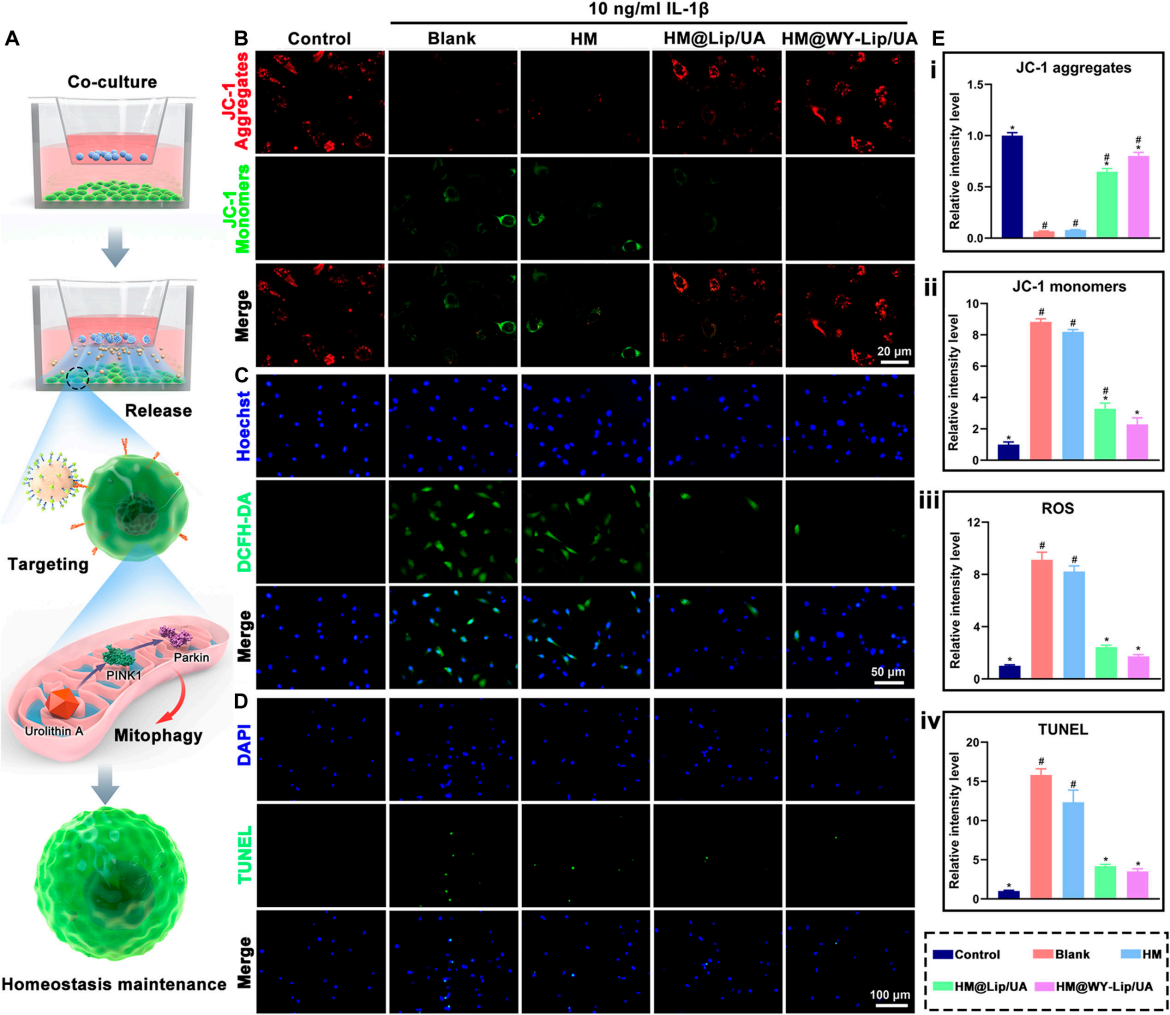
Evaluation of mitochondrial function restoration, ROS scavenging and cell survival rescue. (A) Scheme illustration of the mechanism of cell homeostasis regulation by HM@WY-Lip/UA. (B) Mitochondrial membrane potential detected by JC-1 assay. (C) ROS generation was assessed by DCFH-DA. (D) TUNEL staining. (E) Quantification analysis of (i) JC-1 aggregates, (ii) JC-1 monomers, (iii) DCFH-DA fluorescence intensity, and (iv) TUNEL fluorescence intensity. *n* = 3 for each group; # and * indicate statistical significance (*P* < 0.05) when comparing with the control and blank groups, respectively.

The pathogenesis of osteoarthritis is closely linked to mitochondrial dysfunction. Mitophagy is essential for eliminating dysfunctional mitochondria, facilitating their replacement with functional and healthy mitochondria, which, in turn, contributes to the maintenance of cellular homeostasis (Fig. 4A). The assessment of mitochondrial function can be effectively conducted through the evaluation of the mitochondrial membrane potential, which serves as a highly responsive indicator [42]. Therefore, in this study, JC-1 staining was employed to detect mitochondrial membrane potential, thereby assessing the restoration of mitochondrial function in chondrocytes following treatment with HM@WY-Lip/UA. As shown in Fig. 4B and E, the blank group demonstrated a statistically significant reduction in mitochondrial membrane potential. This revealed that the exposure of chondrocytes to IL-1β resulted in a significant impairment of mitochondrial function. This finding is consistent with previous literature in which IL-1β, as a common inflammatory cytokine, negatively regulates mitochondrial respiration in chondrocytes, leading to cellular mitochondrial dysfunction [43]. In addition, no statistically significant disparity in fluorescence intensity was identified between the HM group and the blank group. This finding implied that the pure HM treatment did not exert a significant influence on the mitochondrial function of the cells. Notably, compared to the blank group, the HM@Lip/UA and HM@WY-Lip/UA groups demonstrated increased red fluorescence intensity and a more significant reduction in JC-1 monomers, particularly in the HM@WY-Lip/UA group. This indicated that the incorporation of UA into composites significantly enhanced the restoration of mitochondrial function, owing to its role in promoting mitophagy. Moreover, the addition of WY peptide enhanced the penetration of liposomes into chondrocytes, thereby improving the efficacy of UA and further contributing to the restoration of mitochondrial function.

### ROS scavenging and cell survival rescue

It is widely acknowledged that a reduction in mitochondrial membrane potential can result in an elevation of mitochondrial-derived ROS, which serves as the primary origin of intracellular ROS [44]. Mitophagy hinders the generation of ROS by enhancing the functionality of mitochondria. In this research, the evaluation of intracellular ROS production was conducted through employing a dichlorodihydrofluorescein diacetate (DCFH-DA) probe to stain chondrocytes (Fig. 4C and E). The findings indicated that the levels of ROS showed a substantial decrease in both the HM@Lip/UA and HM@WY-Lip/UA groups in comparison to the blank and HM groups. Furthermore, the HM@WY-Lip/UA group exhibited a further reduction in ROS levels compared to the HM@Lip/UA group. In accordance with JC-1 staining, exposure to IL-1β also elicited an augmentation in ROS production within chondrocytes. The delivery of UA resulted in a reduction of ROS production through the augmentation of mitophagy, a phenomenon that was further intensified by the addition of WY. ROS fulfills important physiological functions, encompassing the regulation of cellular proliferation and differentiation, adaptation to hypoxic conditions, and the regulation of autophagy and immunity. Nevertheless, excessive ROS production leads to organelle impairment, cellular death, and the initiation of pro-inflammatory reactions that contribute to the pathogenesis and progression of numerous diseases [45]. The experimental findings of this study have substantiated that HM@WY-Lip/UA possessed the capability to induce mitophagy, consequently leading to a reduction in ROS production.

Mitophagy serves as a defensive mechanism that hinders apoptosis and mitigates the progression of osteoarthritis to a certain degree. In order to assess the rescue effects of HM@WY-Lip/UA on the survival of damaged chondrocytes, the apoptosis in chondrocytes was investigated through terminal deoxynucleotidyl transferase dUTP nick end labeling (TUNEL) staining (Fig. 4D and E). The findings from the TUNEL assay revealed that the HM@Lip/UA and HM@WY-Lip/UA groups demonstrated a lower quantity of TUNEL-positive cells compared to blank and HM groups. In particular, HM@Lip/UA significantly maintained chondrocyte homeostasis and inhibited apoptosis, and HM@WY-Lip/UA further augmented this protective effect. In accordance with previous findings, mitophagy serves as a safeguard for preserving the homeostasis and operational efficiency of the mitochondria during cellular injury and death [46]. An increasing body of evidence indicates a robust correlation between mitophagy and apoptosis [47]. Additionally, ROS are acknowledged as signaling molecules that are produced in reaction to various apoptotic stimuli, and they evidently contribute to the activation of CaMKII-mediated apoptosis. The ROS scavenging serves as an important mechanism through which mitophagy effectively suppresses apoptosis [48]. In this investigation, the suppression of ROS production exhibited a notable correlation with the cell survival rescue. Therefore, our findings provided confirmation that HM@WY-Lip/UA possesses the ability to promote ROS scavenging and cell survival rescue.

### Effect of cellular homeostasis maintenance

**Fig. 5.**
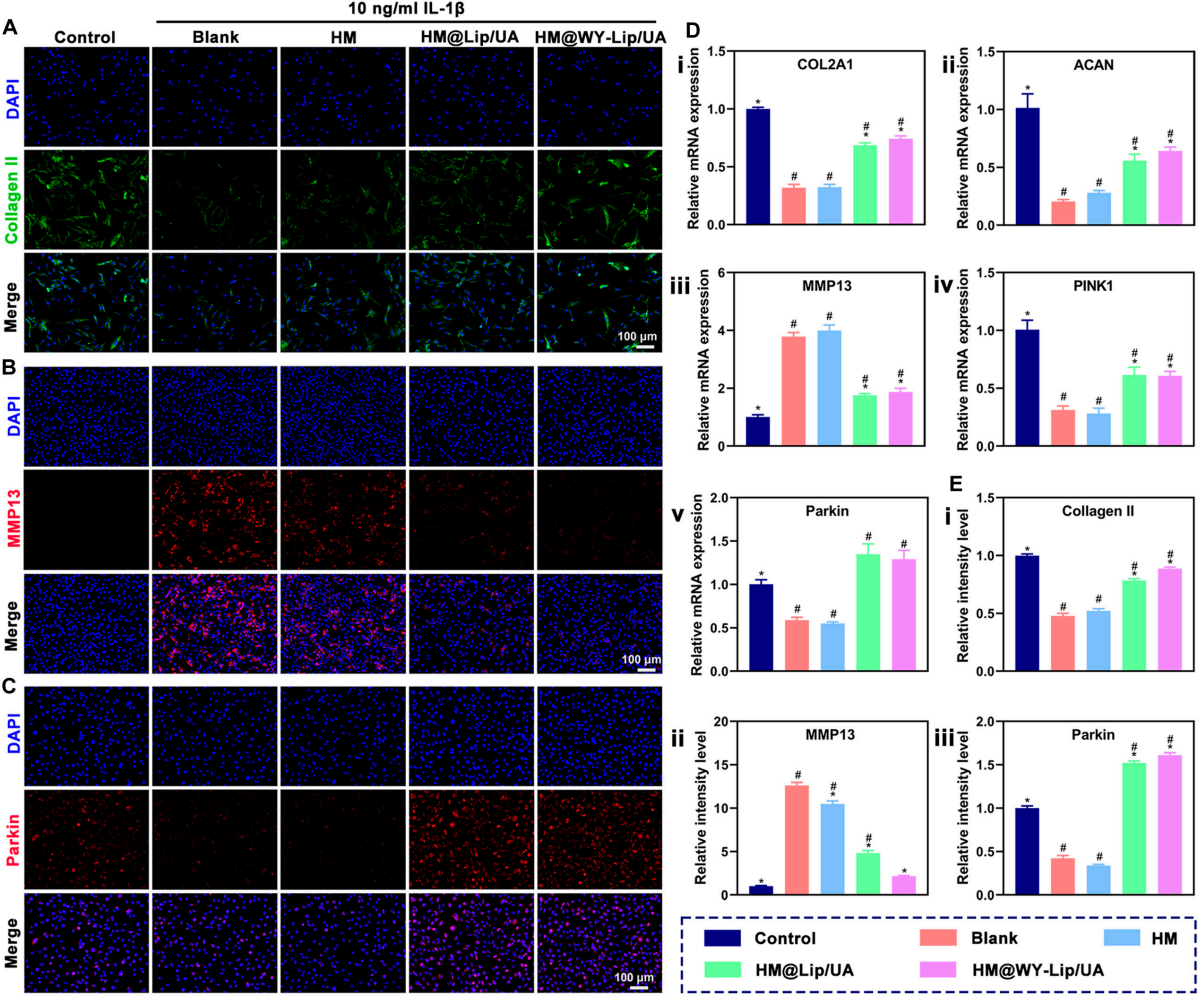
Examination of cellular homeostasis maintenance. (A) Immunofluorescence analysis of Collagen II. (B) Immunofluorescence analysis of MMP13. (C) Immunofluorescence analysis of Parkin. (D) mRNA expression of (i) COL2A1, (ii) ACAN, (iii) MMP13, (iv) PINK1, and (v) Parkin. (E) The relative immunofluorescence intensity of (i) Collagen II, (ii) MMP13, and (iii) Parkin. *n* = 3 for each group; # and * indicate statistical significance (*P* < 0.05) when comparing with the control and blank groups, respectively.

During osteoarthritis progression, dysfunctional mitochondria can generate ROS, and the subsequent accumulation of ROS in turn further aggravates oxidative stress and mitochondrial damage [49]. Furthermore, mitochondrial dysfunction can influence the structure and composition of cartilage ECM, which eventually contributes to cartilage degradation [50]. In this study, quantitative real-time polymerase chain reaction (qRT-PCR) and immunofluorescence were conducted to assess the efficacy of HM@WY-Lip/UA on chondrocyte degeneration at both gene and protein expression levels. Based on the findings depicted in Fig. 5D, the blank group showed that the mRNA expression of MMP13 exhibited a significant increase, whereas the expression of COL2A1, ACAN, PINK1, and Parkin exhibited significant decreases compared to the control group. The treatment with pure HM had no significant effect on the regulation of mRNA expression in each marker. However, HM@WY-Lip/UA presented a strong ability to inhibit the MMP13 mRNA levels and promote the mRNA levels of COL2A1, ACAN, PINK1, and Parkin. These results demonstrated that the HM@WY-Lip/UA promoted mitophagy by delivering UA, leading to increased ECM secretion and reduced MMP13 production. In accordance with the findings from qRT-PCR, immunofluorescence showed that the protein expression of Collagen II and Parkin were significantly upregulated by the treatment with HM@WY-Lip/UA, while the protein expression of MMP13 was suppressed (Fig. 5A to C and E). In summary, these results revealed that HM@WY-Lip/UA had an advantage in directing cellular homeostasis maintenance on chondrocytes.

### Therapeutic effect of osteoarthritis in vivo

**Fig. 6.**
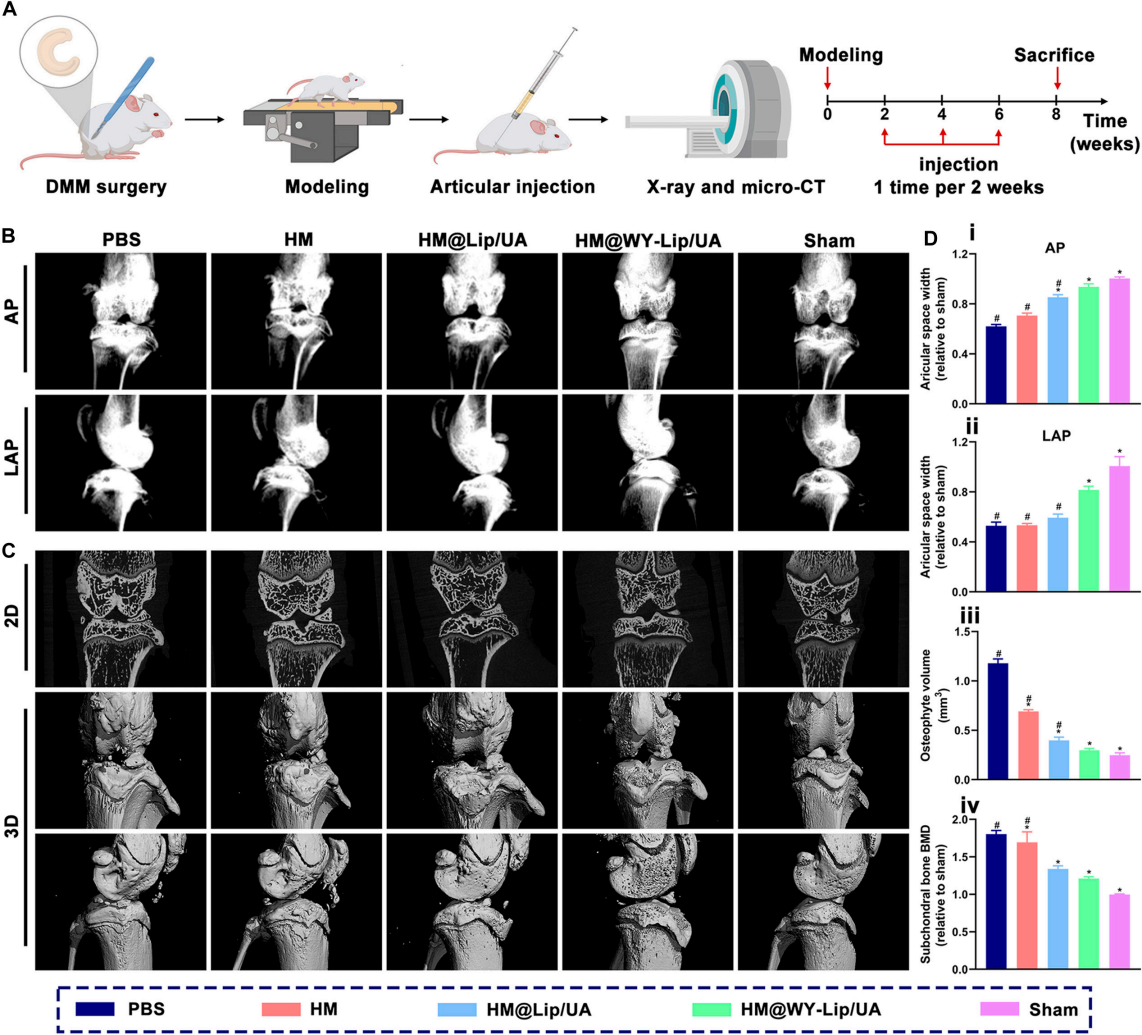
Radiological examination of HM@WY-Lip/UA for osteoarthritis treatment. (A) Schematic diagram of the animal experiments. (B) Representative x-ray images. (C) Representative micro-CT images. (D) Quantitative analysis of (i) JSW from x-ray in anterior–posterior view, (ii) JSW from x-ray in lateral view, (iii) osteophyte volume, and (iv) subchondral bone density. *n* = 5 for each group; # and * indicate statistical significance (*P* < 0.05) when comparing with the sham and PBS groups, respectively.

The disruption of medial meniscus stability, with high clinical relevance, has been extensively employed for establishing animal models of osteoarthritis. The mechanical stability of the knee joint can be disrupted by the transection of the medial meniscotibial ligaments, thereby causing cartilage injury. These changes closely resemble some of the processes observed in human osteoarthritis progress [51]. In this work, rats were randomly divided into sham, phosphate buffered saline (PBS), HM, HM@Lip/UA, and HM@WY-Lip/UA groups to evaluate the therapeutic efficacy in osteoarthritic degeneration in vivo (Fig. 6A). According to the x-ray imaging, a notable reduction in joint space widths (JSWs) was observed in the PBS, HM, and HM@Lip/UA groups in comparison to the sham group. Conversely, the HM@WY-Lip/UA group exhibited a notable enhancement in JSW, with no significant difference compared to sham group (Fig. 6B and D). Next, micro-CT scanning was performed to further analyze the characteristics of osteoarthritis. Osteophyte formation and subchondral bone sclerosis are commonly used parameters to assess the severity of osteoarthritis by micro-CT [52]. As depicted in Fig. 6C and D, a substantial increase in the development of osteophytes was found in the PBS, HM, and HM@Lip/UA groups. Conversely, the HM@WY-Lip/UA group produced only a minimal amount of osteophyte, even close to the sham group. In terms of subchondral osteosclerosis, the HM@WY-Lip/UA group exhibited the lowest levels compared to the other experimental groups. These results indicated that the joint destruction and remodeling in osteoarthritis could be relieved through intra-articular injection of HM@WY-Lip/UA.

**Fig. 7.**
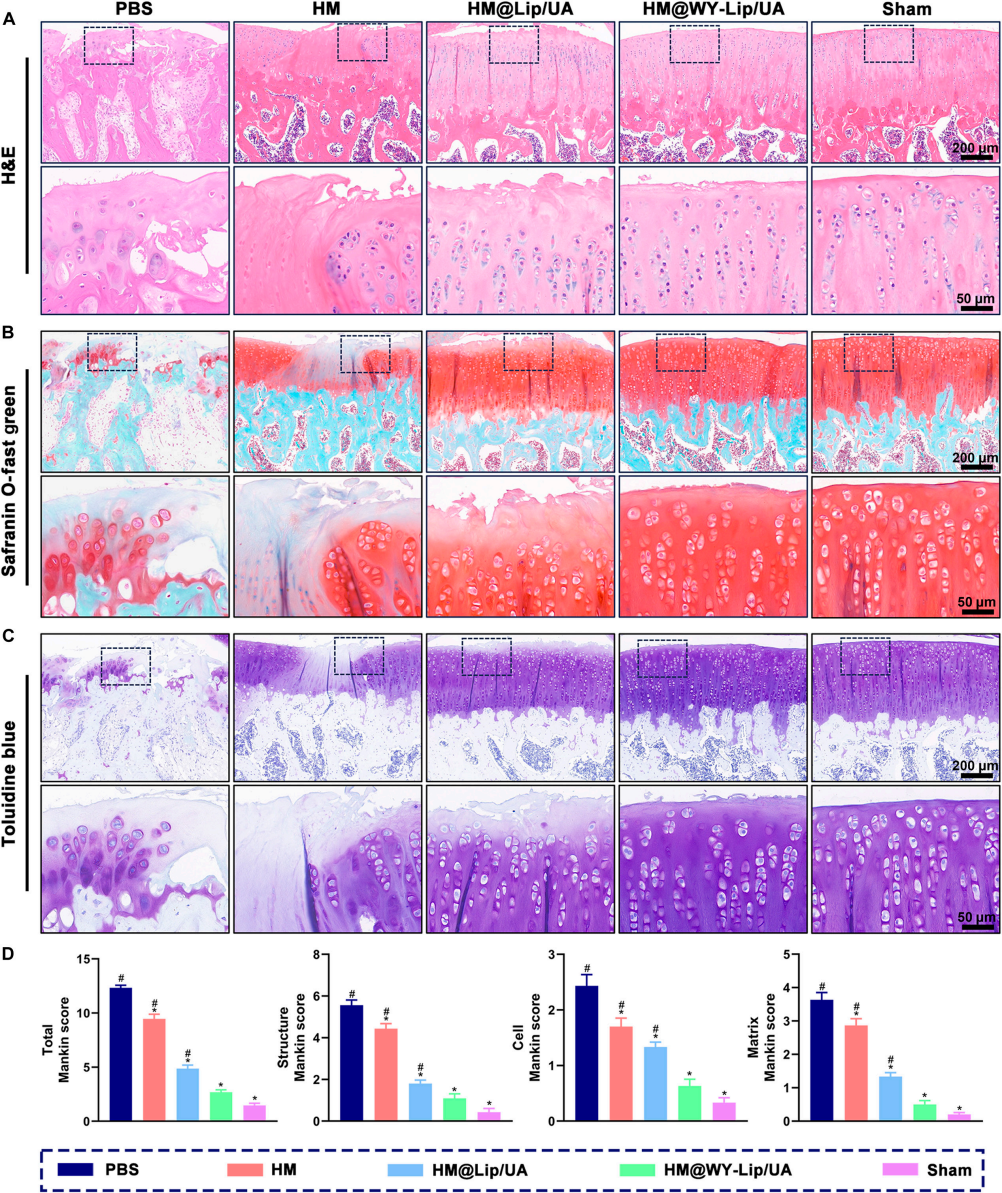
Histological staining. (A) H&E staining. (B) Safranin O-fast green staining. (C) Toluidine blue staining. (D) Mankin scores of articular cartilage. *n* = 5 for each group; # and * indicate statistical significance (*P* < 0.05) when comparing with the sham and PBS groups, respectively.

Subsequently, the analysis of tissue sections from hearts, livers, spleens, lungs, and kidneys, which were stained with hematoxylin and eosin (H&E), revealed no significant histological changes in all experimental groups when compared to the sham group. These findings indicated that the intra-articular injection of HM@WY-Lip/UA was quite safe (Fig. S4). In addition, histological staining was also performed to assess cellular, structural, and ECM changes in articular cartilage (Fig. S5). As indicated in Fig. 7A to D, the cartilage in the sham group showed a natural and uniform structure with a strong positive intensity of safranin O-fast green and toluidine blue staining. Conversely, the PBS group demonstrated significant erosion in the cartilage surface, disorganized chondrocytes, and weak positive staining. Although HM and HM@Lip/UA groups also showed degenerative changes, surface abrasion, and cartilage matrix degradation compared with sham group, they had a substantial decline in the Mankin scores compared with the PBS group. In comparison, treatment with HM@WY-Lip/UA presented the most favorable outcomes in terms of maintaining the normal articular cartilage structure and alleviating the degeneration.

**Fig. 8.**
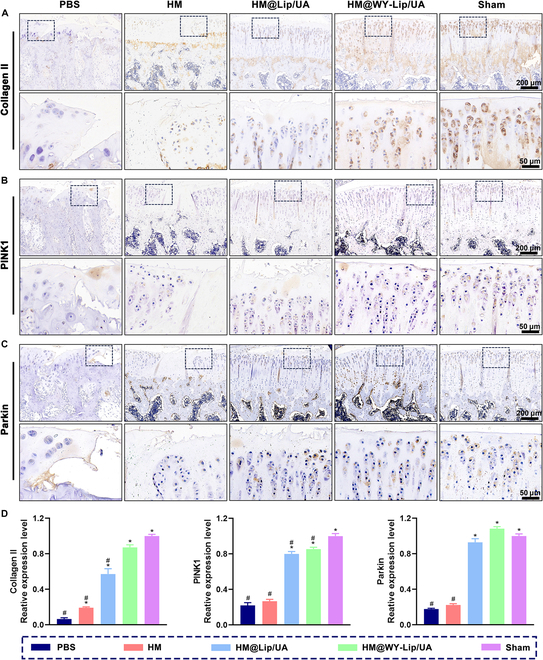
Immunohistochemistry staining. (A) Collagen II staining. (B) PINK1 staining. (C) Parkin staining. (D) Quantitative analysis of protein expression of Collagen II, PINK1, and Parkin. *n* = 5 for each group; # and * indicate statistical significance (*P* < 0.05) when comparing with the sham and PBS groups, respectively.

In the destabilization of the medial meniscus (DMM) rat model, osteoarthritis development is characterized by a gradual decline in the proliferation of chondrocytes and the synthesis of ECM, which can be observed through immunohistochemical analysis revealing a reduction in the expression of Collagen II [53]. Therefore, immunohistochemical analyses were performed to evaluate the protein levels of Collagen II (Fig. S6 and Fig. 8A and D). Compared with the sham group, Collagen II protein abundance was decreased in the PBS, HM, and HM@Lip/UA groups. However, there was no statistical difference between HM@WY-Lip/UA and sham groups. In addition, as illustrated in Fig. S6 and Fig. 8B to D, immunohistochemical staining also provided confirmation that the protein levels of PINK1 and Parkin were significantly declined in the PBS group, indicating the DMM-induced suppression of mitophagy in rat osteoarthritic cartilage tissue. These findings align with previous research that illustrated a reduction in mitophagy and the adverse impact of oxidative stress on chondrocytes in the DMM animal model [54]. However, these decreases were reversed by administration based on UA delivery, especially the HM@WY-Lip/UA. It clearly indicated the effect of HM@WY-Lip/UA on promoting mitophagy in vivo, which was consistent with the outcomes observed in in vitro experiments. In summary, these findings suggested that the injection of HM@WY-Lip/UA could alleviate cartilage matrix degradation through activation of mitophagy with chondrocyte targeting, which exhibits promising therapeutic potential in the treatment of osteoarthritis.

## Conclusion

This study aimed to investigate the potential benefits and practicality of employing a mitochondria-oriented strategy for the management of osteoarthritis. We found that WY-decorated liposomes can efficiently target chondrocytes and facilitate cellular uptake. With the integration of HMs prepared by microfluidic technology, mitochondria-oriented HMs not only sustain the release of UA but also target the mitochondria dysfunctional microenvironment, facilitating chondrocyte metabolic homeostasis at a subcellular level. To sum up, this subcellular therapeutic platform is highly efficient, and its feasibility for mass production makes it promising for clinical translation in anti-aging therapy such as osteoarthritis.

## Materials and Methods

### Preparation and characterization of liposomes

The synthesis of DSPE-PEG-WY was conducted initially. DSPE-PEG2K-NHS (100 mg) underwent dissolution in 3 ml of dimethylformamide, followed by the addition of WY peptide (1.1 eq.) and triethylamine (3.0 eq.) to the system, and the reaction was conducted for 12 h at ambient temperature. The solution was subjected to dialysis for 24 h and freeze-dried to acquire purified DSPE-PEG-WY. ^1^H NMR (Bruker, Germany) was used to determine the successful synthesis of DSPE-PEG-WY. Following this, the thin-film dispersion technique was utilized to prepare WY-Lip/UA. Briefly, a mixture of lecithin, cholesterol, DSPE-PEG-WY, and UA (in a ratio of 4:1:0.1:0.2 w/w) was dissolved in chloroform and subsequently transformed into thin films using a round-bottomed flask in a rotary evaporator operating at 50 r/min and 40 °C. Subsequently, the films underwent hydration using preheated PBS at a temperature of 40 °C and a rotational speed of 50 r/min. The obtained products underwent sonication and subsequent extrusion using a liposome extruder (Avanti, USA) equipped with polycarbonate membrane filters (Avanti, USA) featuring pore sizes of 450 nm and 220 nm, respectively, for 10 cycles each. Ultimately, WY-Lip/UA were successfully obtained. Lip and Lip/UA were also synthesized employing the identical procedure.

To comprehensively characterize the preparation of liposomes, morphological analysis of the liposomes was conducted utilizing a TEM (FEI Talos L120C, USA). The samples were prepared through the application of a small quantity of a diluted liposome suspension onto a copper mesh that had been coated with a carbon layer. TEM images were acquired using an accelerating voltage of 200 kV at room temperature. Moreover, the determination of liposome particle size and zeta potential was conducted utilizing dynamic light scattering (DLS) with the employment of a Zetasizer (Malvern Nano-ZS, UK). Concisely, the liposome samples were diluted at a ratio of 1:100 in a 5% glucose solution and subsequently analyzed using DLS, employing a suitable polystyrene cuvette.

### Synthesis of HAMA

Synthesis of HAMA was carried out based on the method reported in the literature [55]. In brief, 2 g of HA (molecular weight = 74 kDa) was completely dissolved using 100 ml of deionized water and reacted with 6 ml of methacrylic anhydride at pH 8.0. A continuous stir was performed for 24 h at a sub-zero temperature on the reaction mixture. Following that, a solution of 0.5-M NaCl was introduced into the aforementioned mixture, which was subsequently subjected to precipitation by doubling the volume of ethanol. The purified product was obtained through 3 days of dialysis with a 3,500-Da molecular weight cutoff. Afterward, the purified product underwent freeze-drying in order to yield HAMA with a spongy texture. ^1^H NMR was used to determine the percentage of methacrylation in HAMA.

### Synthesis and characterization of mitochondrial-oriented HM@WY-lip/UA

HM@WY-Lip/UA were fabricated through the generation of water-in-oil droplets using microfluidics [56]. The water phase consisted of a 5 wt% mixture of HAMA, WY-Lip/UA, and a photoinitiator (in a ratio of 80:12:8, w/w/w). Additionally, a mixture consisting of paraffin oil and 5 wt% Span 80 was formulated for the oil phase. The oil and water phases were combined and introduced into the microfluidic device through the inlet utilizing a syringe pump, using a flow rate ratio that had been adjusted appropriately. Cross-linking was achieved by exposing the droplets to UV radiation for 5 min. After this, the cross-linked microspheres underwent a cleansing process involving acetone and deionized water, followed by a freeze-drying period of 48 h.

Bright-field microscopy (ZEISS, Germany) was used to examine the morphology and size of HM@WY-Lip/UA. To validate the effective binding of liposomes to HM, liposomes labeled with DiI were used to confirm the successful incorporation of liposomes, which were subsequently observed utilizing LSCM. After 60 s of gold spraying using an ion sputtering instrument, the surface morphology and microstructure of HM or HM@WY-Lip/UA were examined through SEM analysis (FEI Sirion 200, USA). SEM images were obtained at an accelerating voltage of 10 kV.

### In vitro degradation test

The evaluation of the biodegradability of HM or HM@WY-Lip/UA was based on enzymatic degradation experiments. The degradation kinetics of the samples were conducted under ambient conditions. Briefly, 30 mg of HM or HM@WY-Lip/UA was immersed in 1,000 U/ml hyaluronidase in PBS and the degradation was observed at 37 °C on a shaker. It was replaced every 3 days with a fresh hyaluronidase solution. After collecting samples at predetermined intervals, excess PBS was eliminated with filter paper. Thereafter, each sample was weighed again and compared to its initial weight. Additionally, it is important to observe and note the alterations occurring in the microspheres.

### Encapsulation efficiency and drug release assay

The microspheres/liposomes with a mass of 1 mg were permitted to undergo complete fragmentation, and the resulting OD value was tested by a UV-5100 spectrophotometer (Metash, China). Specifically, the encapsulation efficiency was computed using the subsequent formula: encapsulation efficiency (%) = (weight of encapsulated UA)/(initial weight of UA added) × 100%. The determination of the release profile of UA was also conducted. The release of UA was detected using a UV spectrophotometer. Concisely, WY-Lip/UA or HM@WY-Lip/UA was immersed in 1 ml of PBS at 37 °C with gentle agitation (80 rpm). The medium of release was collected at different time intervals for UV analysis and subsequently substituted with 1 ml of fresh PBS. The determination of the drug release rate involved calculating the ratio between the amount of UA released and the total quantity of loaded drugs.

### Isolation and culture of chondrocytes

Chondrocytes were isolated from neonatal Sprague–Dawley (SD) rats. In summary, the rats were euthanized and subsequently immersed in a solution of 70% ethanol for a duration of 15 min. The knee articular cartilage of the rat was harvested utilizing sterilized scissors and forceps. Cartilage was initially cleansed 3 times using PBS and then fragmented into smaller sections. These fragmented cartilage slices were subsequently subjected to sequential digestion, first in a 0.25% trypsin solution for 5 min, followed by digestion in DMEM medium supplemented with 10% type II collagenase for a period of 6 h. The primary chondrocytes were acquired and subsequently introduced into culture flasks, with the medium being replaced every 3 days. Chondrocytes were passaged at a 1:3 ratio when they reached 80% confluence.

### Biocompatibility test

Chondrocyte uptake of liposomes was observed using liposomes labeled with DiI in HM@Lip/UA or HM@WY-Lip/UA, to illustrate the effect of WY on chondrocyte targeting. Chondrocytes were introduced onto the lower compartment of transwell, while the upper layer was inoculated with DiI-labeled HM@Lip/UA or HM@WY-Lip/UA. Following a period of 24 to 48 h of co-culturing with chondrocytes in an environment devoid of light, PBS was used to eliminate unbound DiI, and the presence of fluorescence within the chondrocytes was detected through the utilization of LSCM.

The cytotoxicity of HM@WY-Lip/UA was evaluated through live-dead staining, while chondrocyte proliferation was evaluated using the CCK-8 assay. Live/dead staining reagent was introduced for 30 min following the manufacturer’s instructions after 1, 3, and 5 days of co-culture. Afterward, fluorescence microscopy was employed to examine the samples, where live cells were identified by their green calcein-acetoxymethyl ester (calcein-AM) staining, while dead cells were distinguished by their red propidium iodide staining. Consequently, the utilization of calcein-AM in conjunction with propidium iodide was employed to achieve simultaneous dual fluorescence staining of viable and non-viable cells. On the other hand, the reduction of water-soluble tetrazolium in cell counting kit-8 (CCK8) solution by mitochondrial dehydrogenase leads to the generation of orange formazan. Consequently, as cellular proliferation increases, the intensity of the dye’s color deepens. The culture medium was supplemented with CCK8 following the instructions provided by the reagent supplier. Next, the chondrocytes were co-incubated with CCK8 for 2 h, and a microplate reader (Molecular Devices, Japan) was used to detect the OD value at 450 nm.

Furthermore, cell morphology was observed through the utilization of 4′,6-diamidino-2-phenylindole (DAPI; blue) and phalloidin (red) staining. In summary, chondrocytes were immobilized using a 4% paraformaldehyde (PFA) solution and the cellular membrane was rendered permeable through the application of a 0.1% Triton X-100 solution. Following that, chondrocytes were concurrently exposed to phalloidin for 30 min at ambient temperature, while being shielded from light. This was followed by staining with DAPI for a period of 10 min under identical conditions. The samples underwent 3 rounds of washing with PBS in order to eliminate any residual fluorescence that was not bound. The nucleus exhibits a blue hue, while the cytoskeleton displays a red hue, and the acquisition of images was conducted using LSCM.

### Determination of mitochondrial membrane potential

To replicate the characteristics of osteoarthritis, chondrocytes affected by osteoarthritis were acquired through a 24-h treatment with IL-1β at a concentration of 10 ng/ml (blank group). Chondrocytes that did not undergo IL-1β treatment were designated as the control group. HM, HM@Lip/UA, or HM@WY-Lip/UA was introduced into the upper chamber of the transwell system to investigate the therapeutic impact of HM@WY-Lip/UA on the cellular homeostasis of osteoarthritis chondrocytes. JC-1 (MedChemExpress, USA), a fluorescent probe, was utilized to evaluate the mitochondrial membrane potential in chondrocytes co-cultured with HM@WY-Lip/UA. An elevated mitochondrial membrane potential induces the accumulation of JC-1 aggregates in the mitochondrial matrix, leading to the creation of a polymer emitting red fluorescence. Conversely, a low membrane potential prevents JC-1 from accumulating in the matrix, causing it to remain in a monomeric state and exhibit green fluorescence. The identification of diminished red fluorescence intensity and heightened green fluorescence intensity typically signifies the existence of mitochondrial dysfunction. JC-1 was incubated with the cells at a temperature of 37 °C, shielded from light, for 30 min. The JC-1 dye was removed, and the chondrocytes underwent 3 washes with PBS buffer. The fluorescence emitted by JC-1 was visualized utilizing LSCM, enabling the imaging of chondrocytes.

### Detection of ROS

The HM@WY-Lip/UA was subjected to co-culture with chondrocytes in a transwell system for a duration of 48 h. Subsequently, ROS was assessed through the utilization of a ROS assay kit (Beyotime, China). Chondrocytes that did not undergo IL-1β treatment were designated as the control group. Following the co-cultivation of chondrocytes, the cells were subjected to staining with the DCFH-DA probe for a duration of 20 min at a temperature of 37 °C, following the guidelines provided by the kit. In particular, the DCFH-DA compound was initially diluted with a serum-free culture solution at 1:1,000, yielding a concentration of 10 μmol/L. Incorporate a suitable quantity of diluted DCFH-DA to encompass the entirety of the cellular population. The entirety of the experiment was conducted under dark conditions. The chondrocytes were subsequently rinsed 3 times using PBS in order to eliminate any remaining unbound fluorescence. The visualization of fluorescence was conducted using LSCM.

### Assessment of cell survival

The cell survival rescue was examined by a TUNEL staining kit (Beyotime, China). The cells underwent a 30-min treatment with 4% PFA, which was subsequently followed by the application of 0.1% Triton X-100 in order to augment the permeability of the cell membrane. Subsequently, the TUNEL assay solution should be prepared in accordance with the provided instructions. It is recommended to add 100 μl of the assay solution to each sample while taking precautions to prevent evaporation of the solution. After this, the nuclei were subjected to DAPI staining under light-restricted conditions for 10 min at room temperature. The specimen was washed using PBS. Eventually, the cells were examined using a fluorescence microscope (excitation wavelength, 488 nm) and assessed for the presence of TUNEL-positive cells.

### Detection of mRNA expression

Following a 48-h treatment of chondrocytes, the quantification of COL2A1, ACAN, MMP13, PINK1, and Parkin mRNA was evaluated using qRT-PCR. Specifically, the RNA Extraction Kit (Thermo Fisher, USA) was employed to isolate total RNA in accordance with the provided instructions. Before performing qRT-PCR, cDNA was obtained utilizing the reverse transcription kit provided by Thermo Fisher. Following this, qRT-PCR was conducted using an ABI 7300 real-time PCR system (ABI, USA). The primer sequences for all genes can be found in Table S1. The normalization of gene expression levels was performed using GAPDH as a reference. The relative expression levels of mRNA were determined by employing the 2^−ΔΔCt^ method. A minimum of 3 repetitions were conducted in the experiments.

### Immunofluorescence staining

Immunofluorescence staining was conducted at ambient temperature to evaluate Collagen II, MMP13, and Parkin protein expression levels in chondrocytes. The cells were subjected to fixation using a 4% PFA solution for 30 min, followed by permeabilization using a PBS solution containing 0.1% Triton X-100 for 10 min. The blocking process involved the utilization of a 2% solution of bovine serum albumin for 30 min. The chondrocytes underwent overnight incubation at 4 °C with rabbit anti-Collagen II, MMP13, and Parkin primary antibodies (Abcam, China) that were diluted in a containment buffer. Subsequently, the chondrocytes were exposed to a 50-min incubation with a suitable secondary antibody. Nuclear staining was achieved through a 10-min incubation with DAPI. The PBS wash was performed 3 times at the end. The excitation wavelength of LSCM was modified in order to acquire the corresponding images of immunofluorescence staining.

### Rat model of osteoarthritis

The conduction of animal studies was carried out subsequent to obtaining approval from the Institutional Animal Care and Use Committee of Chongqing Medical University. Osteoarthritis associated with mechanical instability was induced in a rat model through the utilization of the DMM technique. Briefly, 8-week-old male SD rats were administered anesthesia using pentobarbital sodium at a dosage of 40 mg/kg, followed by the implementation of the DMM surgical procedure. This involved making an incision in the medial meniscus-tibial ligament, which was responsible for attaching the medial meniscus to the tibial plateau. Rats that adhered to an identical protocol for sham surgery were utilized as control subjects, with the sole distinction being the absence of any dissection of the medial meniscus-tibial ligament. Intra-articular injection drug delivery interventions were conducted at intervals of 2 weeks, specifically at 2, 4, and 6 weeks post-surgery. Osteoarthritic rats were allocated into 4 subgroups using randomization, achieved by administering intra-articular injections of 30 μl of PBS, HM, HM@Lip/UA, or HM@WY-Lip/UA, respectively. At the conclusion of the 8-week postoperative period, the rats were humanely euthanized for further analysis.

### Radiographic evaluation

X-ray examination was conducted on the knee joints using an x-ray machine (Faxitron, USA) during the eighth week after the surgical procedure, preceding the euthanasia of the rats. Assess alterations in the JSW by means of both anterior–posterior and lateral views. The isolated knee underwent further analysis using micro-CT (SCANCO, Switzerland). Three-dimensional (3D) images were obtained for the purpose of determining the osteophyte volume and subchondral bone BMD.

### Histologic and immunohistochemical analysis

Rat knee joints were procured, subsequently preserved with a 4% PFA solution, and subjected to a decalcification process in 10% EDTA solution lasting 1 month. Afterward, paraffin embedding was conducted to generate sequential, sagittal pathology sections with a thickness of 5 μm. The sections were analyzed to assess the morphology and matrix of chondrocytes through the application of H&E, safranin o-fast green, and toluidine blue staining. The sections underwent evaluation based on the Mankin scoring system, which assesses cartilage degeneration. This assessment was carried out by observers in a double-blind manner. The Mankin score was recorded for each slice, and subsequently, the average of all scores was computed.

In the context of immunohistochemical staining, the sections were subjected to treatment with a 5% BSA solution for 1 h at ambient temperature. Afterward, they were subjected to an overnight incubation at a temperature of 4 °C with rabbit polyclonal antibodies targeting Collagen II, PINK1, or Parkin. Following the TBST wash, sections were exposed to a secondary antibody for 1 h, and the visualization of immunohistochemical staining was achieved through the utilization of the 3,3′-diaminobenzidine substrate. The quantification of Collagen II, PINK1, and Parkin expression levels was performed utilizing the ImageJ software.

### Statistical analysis

Statistical analysis was performed utilizing SPSS 26.0 software (IBM, USA). The Student *t* test was employed to conduct a comparative analysis of experimental data between 2 groups. For comparing experimental data between multiple groups, either 1-way or 2-way analysis of variance was employed. *P* < 0.05 was considered to indicate statistical significance.

## Data Availability

All data needed to evaluate the conclusions in the paper are present in the paper and/or the Supplementary Materials.

## References

[1] Pigeolet M, Jayaram A, Park KB, Meara JG. Osteoarthritis in 2020 and beyond. Lancet. 2021;397(10270):1059–1060. 33743863 10.1016/S0140-6736(21)00208-7

[2] Borcherding N, Brestoff JR. The power and potential of mitochondria transfer. Nature. 2023;623(7986):283–291. 37938702 10.1038/s41586-023-06537-z PMC11590279

[3] Richard D, Liu Z, Cao J, Kiapour AM, Willen J, Yarlagadda S, Jagoda E, Kolachalama VB, Sieker JT, Chang GH, et al. Evolutionary selection and constraint on human knee chondrocyte regulation impacts osteoarthritis risk. Cell. 2020;181(2):362–381 e328. 32220312 10.1016/j.cell.2020.02.057 PMC7179902

[4] Victorelli S, Salmonowicz H, Chapman J, Martini H, Vizioli MG, Riley JS, Cloix C, Hall-Younger E, Machado Espindola-Netto J, Jurk D, et al. Apoptotic stress causes mtDNA release during senescence and drives the SASP. Nature. 2023;622(7983):627–636. 37821702 10.1038/s41586-023-06621-4 PMC10584674

[5] Mahmoudian A, Lohmander LS, Mobasheri A, Englund M, Luyten FP. Early-stage symptomatic osteoarthritis of the knee—Time for action. Nat Rev Rheumatol. 2021;17(10):621–632. 34465902 10.1038/s41584-021-00673-4

[6] Wang S, Long H, Hou L, Feng B, Ma Z, Wu Y, Zeng Y, Cai J, Zhang DW, Zhao G. The mitophagy pathway and its implications in human diseases. Signal Transduct Target Ther. 2023;8:304. 37582956 10.1038/s41392-023-01503-7 PMC10427715

[7] Gan ZY, Callegari S, Cobbold SA, Cotton TR, Mlodzianoski MJ, Schubert AF, Geoghegan ND, Rogers KL, Leis A, Dewson G, et al. Activation mechanism of PINK1. Nature. 2022;602(7896):328–335. 34933320 10.1038/s41586-021-04340-2 PMC8828467

[8] Xia X, Liu Y, Lu Y, Liu J, Deng Y, Wu Y, Hou M, He F, Yang H, Xu Y, et al. Retuning mitochondrial apoptosis/mitophagy balance via SIRT3-energized and microenvironment-modulated hydrogel microspheres to impede osteoarthritis. Adv Healthc Mater. 2023;12(32):e2302475. 37696643 10.1002/adhm.202302475

[9] Ryu D, Mouchiroud L, Andreux PA, Katsyuba E, Moullan N, Nicolet-dit-Félix AA, Williams EG, Jha P, Lo Sasso G, Huzard D, et al. Urolithin A induces mitophagy and prolongs lifespan in *C. elegans* and increases muscle function in rodents. Nat Med. 2016;22(8):879–888. 27400265 10.1038/nm.4132

[10] Luan P, D’Amico D, Andreux PA, Laurila PP, Wohlwend M, Li H, Imamura de Lima T, Place N, Rinsch C, Zanou N, et al. Urolithin A improves muscle function by inducing mitophagy in muscular dystrophy. Sci Transl Med. 2021;13(588):eabb0319. 33827972 10.1126/scitranslmed.abb0319

[11] Savi M, Bocchi L, Mena P, Dall’Asta M, Crozier A, Brighenti F, Stilli D, del Rio D. In vivo administration of urolithin A and B prevents the occurrence of cardiac dysfunction in streptozotocin-induced diabetic rats. Cardiovasc Diabetol. 2017;16(1):80. 28683791 10.1186/s12933-017-0561-3 PMC5501434

[12] Andreux PA, Blanco-Bose W, Ryu D, Burdet F, Ibberson M, Aebischer P, Auwerx J, Singh A, Rinsch C. The mitophagy activator urolithin A is safe and induces a molecular signature of improved mitochondrial and cellular health in humans. Nat Metab. 2019;1(6):595–603. 32694802 10.1038/s42255-019-0073-4

[13] Fang EF, Hou Y, Palikaras K, Adriaanse BA, Kerr JS, Yang B, Lautrup S, Hasan-Olive MM, Caponio D, Dan X, et al. Mitophagy inhibits amyloid-beta and tau pathology and reverses cognitive deficits in models of Alzheimer's disease. Nat Neurosci. 2019;22(3):401–412. 30742114 10.1038/s41593-018-0332-9 PMC6693625

[14] Singh A, D’Amico D, Andreux PA, Fouassier AM, Blanco-Bose W, Evans M, Aebischer P, Auwerx J, Rinsch C. Urolithin A improves muscle strength, exercise performance, and biomarkers of mitochondrial health in a randomized trial in middle-aged adults. Cell Rep Med. 2022;3(5): Article 100633. 35584623 10.1016/j.xcrm.2022.100633 PMC9133463

[15] Feng K, Ge Y, Chen Z, Li X, Liu Z, Li X, Li H, Tang T, Yang F, Wang X. Curcumin inhibits the PERK-eIF2α-CHOP pathway through promoting SIRT1 expression in oxidative stress-induced rat chondrocytes and ameliorates osteoarthritis progression in a rat model. Oxidative Med Cell Longev. 2019;2019: Article 8574386. 10.1155/2019/8574386 PMC6541984 31223428

[16] Ma T, Chen H, Ruan H, Lv L, Yu Y, Jia L, Zhao J, Li X, Zang Y, Xu X, et al. Natural product, bilobalide, improves joint health in rabbits with osteoarthritis by anti-matrix degradation and antioxidant activities. Front Vet Sci. 2022;9:1034623. 36337189 10.3389/fvets.2022.1034623 PMC9631767

[17] Hecht JT, Veerisetty AC, Wu J, Coustry F, Hossain MG, Chiu F, Gannon FH, Posey KL. Primary osteoarthritis early joint degeneration induced by endoplasmic reticulum stress is mitigated by resveratrol. Am J Pathol. 2021;191(9):1624–1637. 34116024 10.1016/j.ajpath.2021.05.016 PMC8420863

[18] Hodgkinson T, Kelly DC, Curtin CM, O'Brien FJ. Mechanosignalling in cartilage: An emerging target for the treatment of osteoarthritis. Nat Rev Rheumatol. 2022;18(2):67–84. 34934171 10.1038/s41584-021-00724-w

[19] Hamley IW, Castelletto V. Small-angle scattering techniques for peptide and peptide hybrid nanostructures and peptide-based biomaterials. Adv Colloid Interf Sci. 2023;318: Article 102959. 10.1016/j.cis.2023.102959 37473606

[20] Chen JY, Xu W, Dai T, Jiao S, Xue X, Jiang J, Li S, Meng Q. Pioglitazone-loaded cartilage-targeted nanomicelles (Pio@C-HA-DOs) for osteoarthritis treatment. Int J Nanomedicine. 2023;18:5871–5890. 37873552 10.2147/IJN.S428938 PMC10590558

[21] Xue S, Zhou X, Sang W, Wang C, Lu H, Xu Y, Zhong Y, Zhu L, He C, Ma J. Cartilage-targeting peptide-modified dual-drug delivery nanoplatform with NIR laser response for osteoarthritis therapy. Bioact Mater. 2021;6(8):2372–2389. 33553822 10.1016/j.bioactmat.2021.01.017 PMC7844135

[22] Kim HR, Cho HB, Lee S, Park JI, Kim HJ, Park KH. Fusogenic liposomes encapsulating mitochondria as a promising delivery system for osteoarthritis therapy. Biomaterials. 2023;302: Article 122350. 37864947 10.1016/j.biomaterials.2023.122350

[23] Lin F, Wang Z, Xiang L, Deng LF, Cui WG. Charge-guided micro/Nano-hydrogel microsphere for penetrating cartilage matrix. Adv Funct Mater. 2021;31(49): Article 2107678.

[24] Shen J, Chen A, Cai Z, Chen Z, Cao R, Liu Z, Li Y, Hao J. Exhausted local lactate accumulation via injectable nanozyme-functionalized hydrogel microsphere for inflammation relief and tissue regeneration. Bioact Mater. 2022;12:153–168. 35310385 10.1016/j.bioactmat.2021.10.013 PMC8897073

[25] Li X, Li X, Yang J, Lin J, Zhu Y, Xu X, Cui W. Living and injectable porous hydrogel microsphere with paracrine activity for cartilage regeneration. Small. 2023;19(17): Article e2207211. 36651038 10.1002/smll.202207211

[26] Kansiz S, Elcin YM. Advanced liposome and polymersome-based drug delivery systems: Considerations for physicochemical properties, targeting strategies and stimuli-sensitive approaches. Adv Colloid Interf Sci. 2023;317: Article 102930. 10.1016/j.cis.2023.102930 37290380

[27] Cheng RY, Liu L, Xiang Y, Lu Y, Deng L, Zhang H, Santos HA, Cui W. Advanced liposome-loaded scaffolds for therapeutic and tissue engineering applications. Biomaterials. 2020;232: Article 119706. 31918220 10.1016/j.biomaterials.2019.119706

[28] Tang Q, Dong M, Xu Z, Xue N, Jiang R, Wei X, Gu J, Li Y, Xin R, Wang J, et al. Red blood cell-mimicking liposomes loading curcumin promote diabetic wound healing. J Control Release. 2023;361:871–884. 37532149 10.1016/j.jconrel.2023.07.049

[29] Vedadghavami A, Wagner EK, Mehta S, He T, Zhang C, Bajpayee AG. Cartilage penetrating cationic peptide carriers for applications in drug delivery to avascular negatively charged tissues. Acta Biomater. 2019;93:258–269. 30529083 10.1016/j.actbio.2018.12.004 PMC6551323

[30] Li X, Dai B, Guo J, Zheng L, Guo Q, Peng J, Xu J, Qin L. Nanoparticle-cartilage interaction: Pathology-based intra-articular drug delivery for osteoarthritis therapy. Nanomicro Lett. 2021;13(1):149. 34160733 10.1007/s40820-021-00670-y PMC8222488

[31] Ranawat A, Guo K, Phillips M, Guo A, Niazi F, Bhandari M, Waterman B. Health economic assessments of hyaluronic acid treatments for knee osteoarthritis: A systematic review. Adv Ther. 2023. 10.1007/s12325-023-02691-y 37899384

[32] Xiao P, Han X, Huang Y, Yang J, Chen L, Cai Z, Hu N, Cui W, Huang W. Reprogramming macrophages via immune cell mobilized hydrogel microspheres for osteoarthritis treatments. Bioact Mater. 2023;32:242–259. 37869722 10.1016/j.bioactmat.2023.09.010 PMC10589729

[33] Daly AC, Riley L, Segura T, Burdick JA. Hydrogel microparticles for biomedical applications. Nat Rev Mater. 2020;5(1):20–43. 34123409 10.1038/s41578-019-0148-6 PMC8191408

[34] Gupta V, Khan Y, Berkland CJ, Laurencin CT, Detamore MS. Microsphere-based scaffolds in regenerative engineering. Annu Rev Biomed Eng. 2017;19:135–161. 28633566 10.1146/annurev-bioeng-071516-044712 PMC11610505

[35] Chang H, Cai F, Zhang Y, Jiang M, Yang X, Qi J, Wang L, Deng L, Cui W, Liu X. Silencing gene-engineered injectable hydrogel microsphere for regulation of extracellular matrix metabolism balance. Small Methods. 2022;6(4): Article e2101201. 34994105 10.1002/smtd.202101201

[36] Chen K, Wang F, Ding R, Cai Z, Zou T, Zhang A, Guo D, Ye B, Cui W, Xiang M. Adhesive and injectable hydrogel microspheres for inner ear treatment. Small. 2022;18(36): Article e2106591. 35106912 10.1002/smll.202106591

[37] Lin IC, Wu JY, Fang CY, Wang SC, Liu YW, Ho ST. Absorption and metabolism of urolithin A and ellagic acid in mice and their cytotoxicity in human colorectal cancer cells. Evid Based Complement Alternat Med. 2023;2023:8264716. 37706115 10.1155/2023/8264716 PMC10497365

[38] Yi S, Zhang C, Hu J, Meng Y, Chen L, Yu H, Li S, Wang G, Zheng G, Qiu Z. Preparation, characterization, and in vitro pharmacodynamics and pharmacokinetics evaluation of PEGylated urolithin A liposomes. AAPS PharmSciTech. 2021;22(1):26. 33404864 10.1208/s12249-020-01890-y

[39] Yang JL, Zhu Y, Wang F, Deng L, Xu X, Cui W. Microfluidic liposomes-anchored microgels as extended delivery platform for treatment of osteoarthritis. Chem Eng J. 2020;400: Article 126004.

[40] Lei Y, Wang Y, Shen J, Cai Z, Zhao C, Chen H, Luo X, Hu N, Cui W, Huang W. Injectable hydrogel microspheres with self-renewable hydration layers alleviate osteoarthritis. Sci Adv. 2022;8(4):eabl6449. 35108047 10.1126/sciadv.abl6449 PMC8809544

[41] Liu WX, Liu A, Li X, Sun Z, Sun Z, Liu Y, Wang G, Huang D, Xiong H, Yu S, et al. Dual-engineered cartilage-targeting extracellular vesicles derived from mesenchymal stem cells enhance osteoarthritis treatment via miR-223/NLRP3/pyroptosis axis: Toward a precision therapy. Bioact Mater. 2023;30:169–183. 37593145 10.1016/j.bioactmat.2023.06.012 PMC10429745

[42] Huang X, Qiu M, Wang T, Li B, Zhang S, Zhang T, Liu P, Wang Q, Qian ZR, Zhu C, et al. Carrier-free multifunctional nanomedicine for intraperitoneal disseminated ovarian cancer therapy. J Nanobiotechnology. 2022;20(1):93. 35193583 10.1186/s12951-022-01300-4 PMC8864853

[43] Qi ZH, Zhu JP, Cai WS, Lou CB, Li ZY. The role and intervention of mitochondrial metabolism in osteoarthritis. Mol Cell Biochem. 2023. 10.1007/s11010-023-04818-9 PMC11224101 37486450

[44] Gong W, Xu J, Wang Y, Min Q, Chen X, Zhang W, Chen J, Zhan Q. Nuclear genome-derived circular RNA circPUM1 localizes in mitochondria and regulates oxidative phosphorylation in esophageal squamous cell carcinoma. Signal Transduct Target Ther. 2022;7:40. 35153295 10.1038/s41392-021-00865-0 PMC8841503

[45] Wang Y, Wang M, Liu Y, Tao H, Banerjee S, Srinivasan S, Nemeth E, Czaja MJ, He P. Integrated regulation of stress responses, autophagy and survival by altered intracellular iron stores. Redox Biol. 2022;55: Article 102407. 35853304 10.1016/j.redox.2022.102407 PMC9294649

[46] Luciani A, Schumann A, Berquez M, Chen Z, Nieri D, Failli M, Debaix H, Festa BP, Tokonami N, Raimondi A, et al. Impaired mitophagy links mitochondrial disease to epithelial stress in methylmalonyl-CoA mutase deficiency. Nat Commun. 2020;11(1):970. 32080200 10.1038/s41467-020-14729-8 PMC7033137

[47] Hu J, Zhang Y, Jiang X, Zhang H, Gao Z, Li Y, Fu R, Li L, Li J, Cui H, et al. ROS-mediated activation and mitochondrial translocation of CaMKII contributes to Drp1-dependent mitochondrial fission and apoptosis in triple-negative breast cancer cells by isorhamnetin and chloroquine. J Exp Clin Cancer Res. 2019;38(1):225. 31138329 10.1186/s13046-019-1201-4 PMC6540563

[48] An X, Ma X, Liu H, Song J, Wei T, Zhang R, Zhan X, Li H, Zhou J. Inhibition of PDGFRβ alleviates endothelial cell apoptotic injury caused by DRP-1 overexpression and mitochondria fusion failure after mitophagy. Cell Death Dis. 2023;14:756. 37980402 10.1038/s41419-023-06272-3 PMC10657461

[49] Sun K, Jing XZ, Guo JC, Yao XD, Guo FJ. Mitophagy in degenerative joint diseases. Autophagy. 2021;17:2082–2092. 32967533 10.1080/15548627.2020.1822097 PMC8496714

[50] Wang XZ, Liu Z, Peng P, Gong Z, Huang J, Peng H. Astaxanthin attenuates osteoarthritis progression via inhibiting ferroptosis and regulating mitochondrial function in chondrocytes. Chem Biol Interact. 2022;366: Article 110148. 36084724 10.1016/j.cbi.2022.110148

[51] Thysen S, Luyten FP, Lories RJ. Targets, models and challenges in osteoarthritis research. Dis Model Mech. 2015;8(1):17–30. 25561745 10.1242/dmm.016881 PMC4283647

[52] Tevlin R, desJardins-Park H, Huber J, DiIorio SE, Longaker MT, Wan DC. Musculoskeletal tissue engineering: Adipose derived stromal cell implementation for the treatment of osteoarthritis. Biomaterials. 2022;286: Article 121544. 35633592 10.1016/j.biomaterials.2022.121544 PMC9267037

[53] Agarwal P, Lee HP, Smeriglio P, Grandi F, Goodman S, Chaudhuri O, Bhutani N. A dysfunctional TRPV4-GSK3beta pathway prevents osteoarthritic chondrocytes from sensing changes in extracellular matrix viscoelasticity. Nat Biomed Eng. 2021;5(12):1472–1484. 33707778 10.1038/s41551-021-00691-3 PMC8433267

[54] Liu L, Zhang W, Liu T, Tan Y, Chen C, Zhao J, Geng H, Ma C. The physiological metabolite alpha-ketoglutarate ameliorates osteoarthritis by regulating mitophagy and oxidative stress. Redox Biol. 2023;62: Article 102663. 36924682 10.1016/j.redox.2023.102663 PMC10026041

[55] Lei YT, Wang Y, Shen J, Cai Z, Zeng Y, Zhao P, Liao J, Lian C, Hu N, Luo X, et al. Stem cell-recruiting injectable microgels for repairing osteoarthritis. Adv Funct Mater. 2021;31(48): Article 2105084.

[56] Li X, Li X, Yang J, du Y, Chen L, Zhao G, Ye T, Zhu Y, Xu X, Deng L, et al. In situ sustained macrophage-targeted nanomicelle-hydrogel microspheres for inhibiting osteoarthritis. Research. 2023;6:0131. 37223475 10.34133/research.0131 PMC10202383

