# Supplementary material for: Mitochondrial-Oriented Injectable Hydrogel Microspheres Maintain Homeostasis of Chondrocyte Metabolism to Promote Subcellular Therapy in Osteoarthritis
Source: Research (Wash D C). 2024 Jan 25;7:0306. doi: 10.34133/research.0306 (PMC10809599; doi:10.34133/research.0306)
Supplement: Supplementary 1 — Figs. S1 to S6 Table S1 [file research.0306.f1.docx]

**Mitochondrial-oriented Injectable Hydrogel Microspheres Maintain Homeostasis of Chondrocyte Metabolism to Promote Subcellular Therapy in Osteoarthritis**

**Authors**

Li Chen^1†^, Jianye Yang^1,2†^, Zhengwei Cai^2†^, Yanran Huang^1,2^, Pengcheng Xiao^1,2^, Hong Chen^1^, Xiaoji Luo^1^, Wei Huang^1*^, Wenguo Cui^2*^, Ning Hu^1*^

**Affiliations**

1. Department of Orthopedics, The First Affiliated Hospital of Chongqing Medical University, Orthopedic Laboratory of Chongqing Medical University, Chongqing 400016, China

2. Department of Orthopaedics, Shanghai Key Laboratory for Prevention and Treatment of Bone and Joint Diseases, Shanghai Institute of Traumatology and Orthopaedics, Ruijin Hospital, Shanghai Jiao Tong University School of Medicine, 197 Ruijin 2nd Road, Shanghai 200025, P. R. China.

^*^Address correspondence to: huangwei68@263.net(W.H.); wgcui80@hotmail.com(W.C.); huncqjoint@yeah.net(N.H.)

^†^ These authors contributed equally to this work.


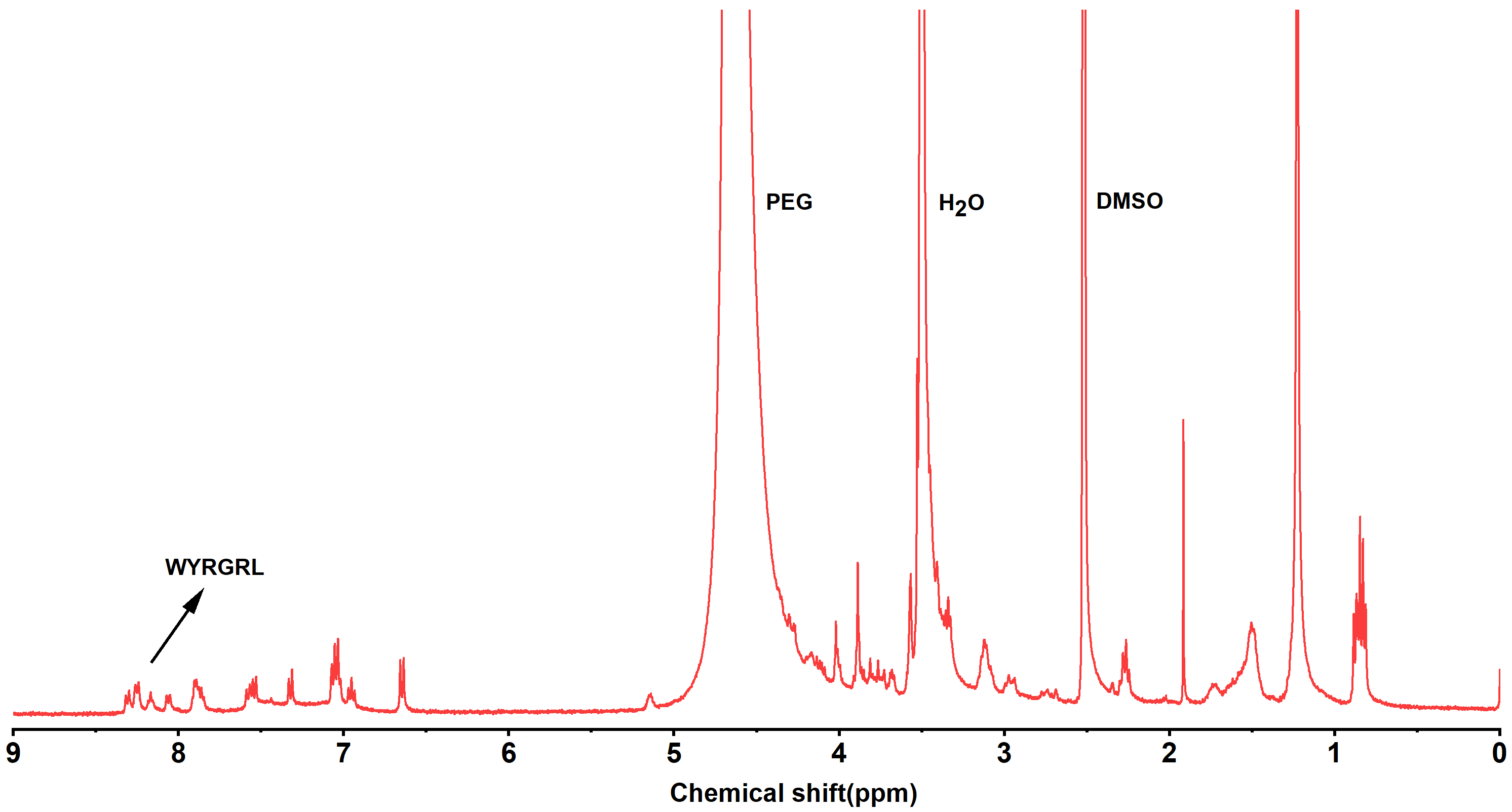


**Fig. S1.** ^1^H-NMR spectrum of DSPE-PEG2K-WYRGRL.


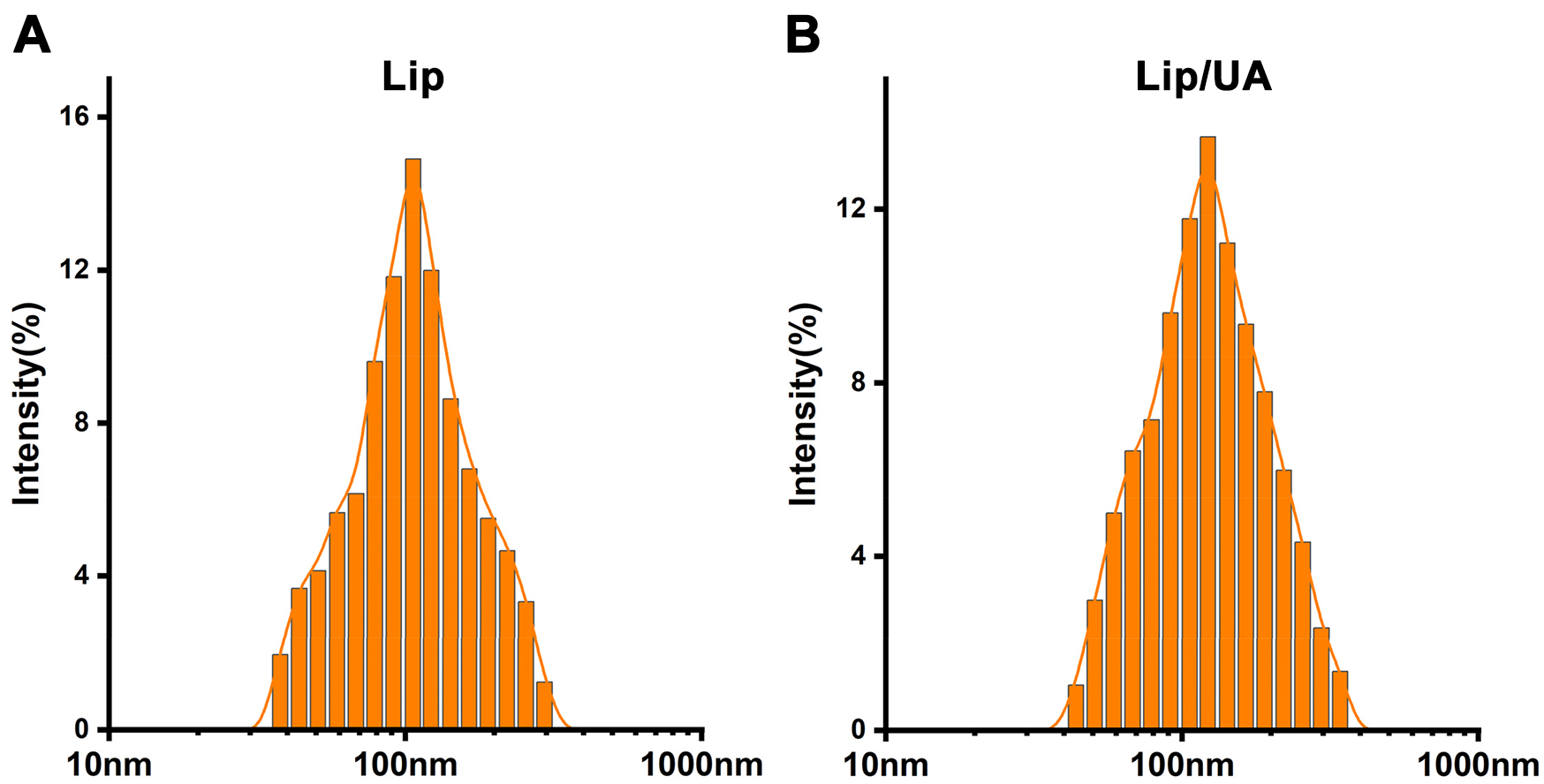


**Fig. S2.** The size distribution of liposomes. (A) Lip. (B) Lip/UA.


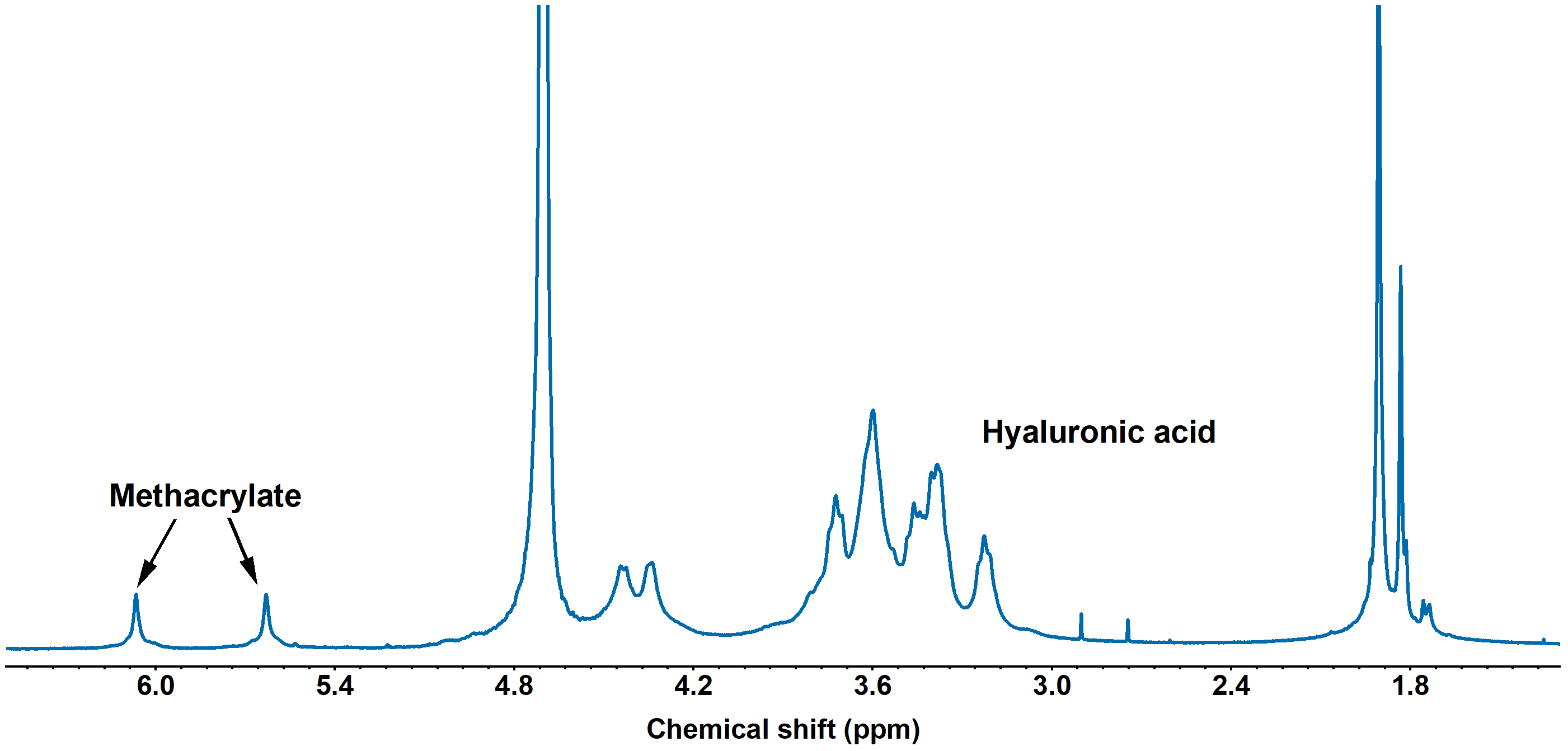


**Fig. S3.** ^1^H-NMR spectrum of HAMA.


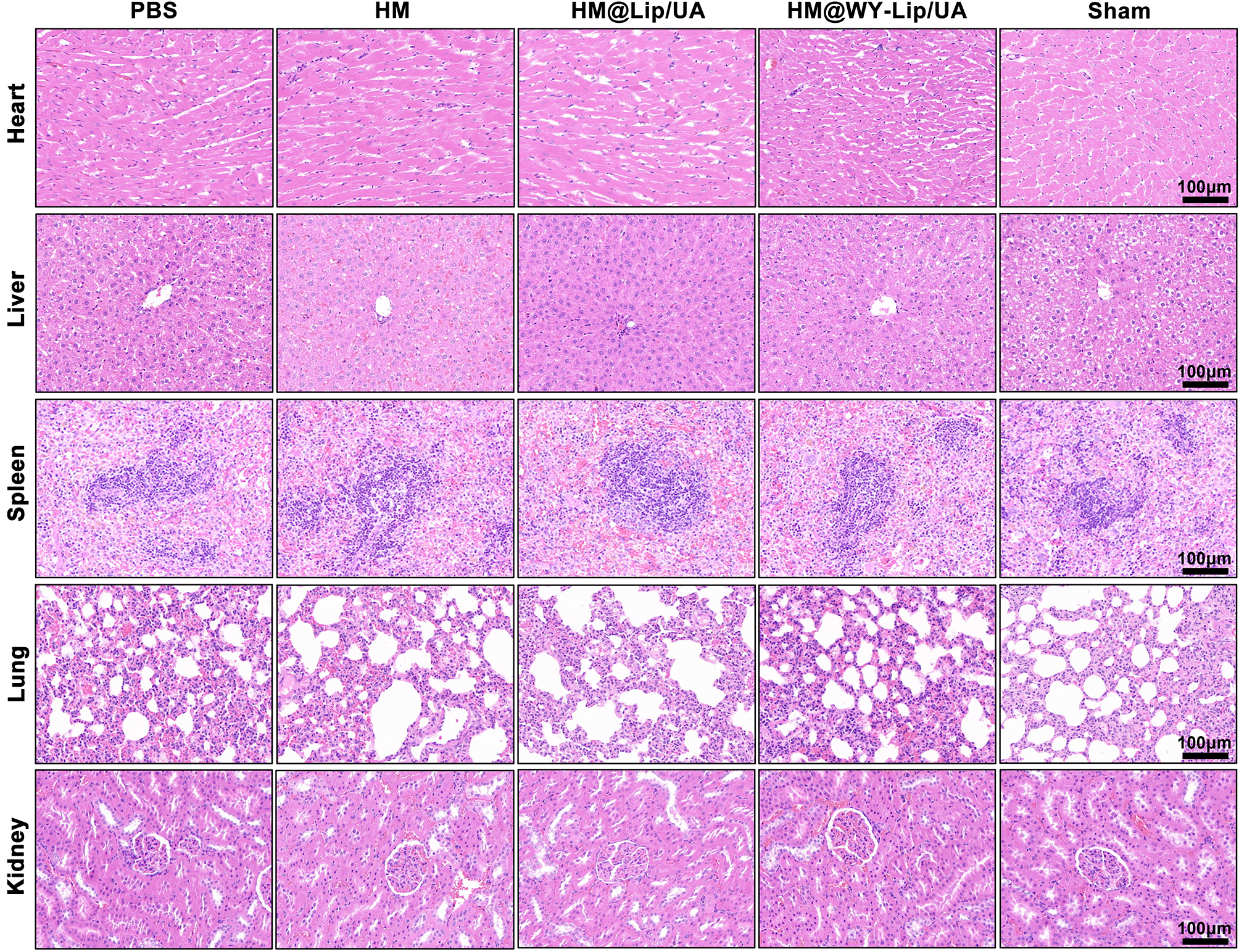


**Fig. S4.** Evaluation of in vivo biocompatibility by H&E staining of the heart, liver, spleen, lung, and kidney tissue.


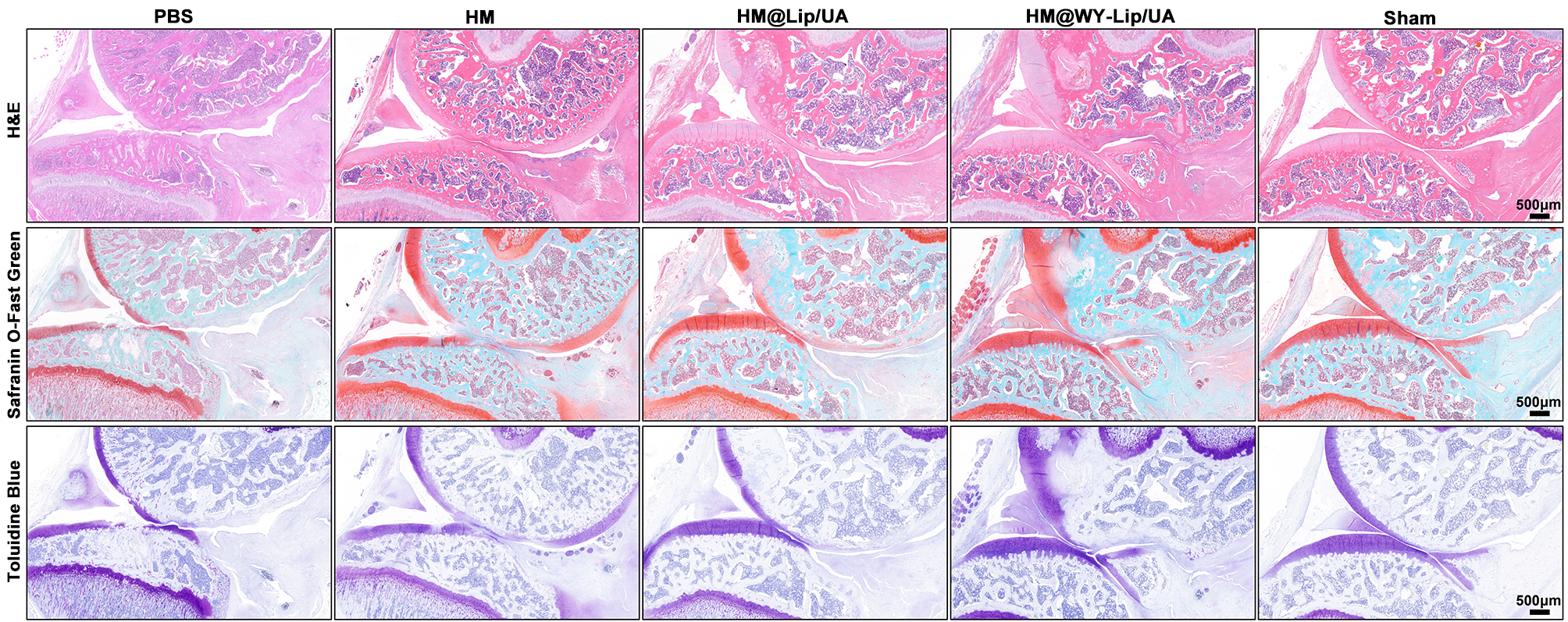


**Figure. S5** The panorama of slices of histological staining in Fig. 7. (A) H&E staining. (B) Safranin O-fast green staining. (C) Toluidine blue staining.


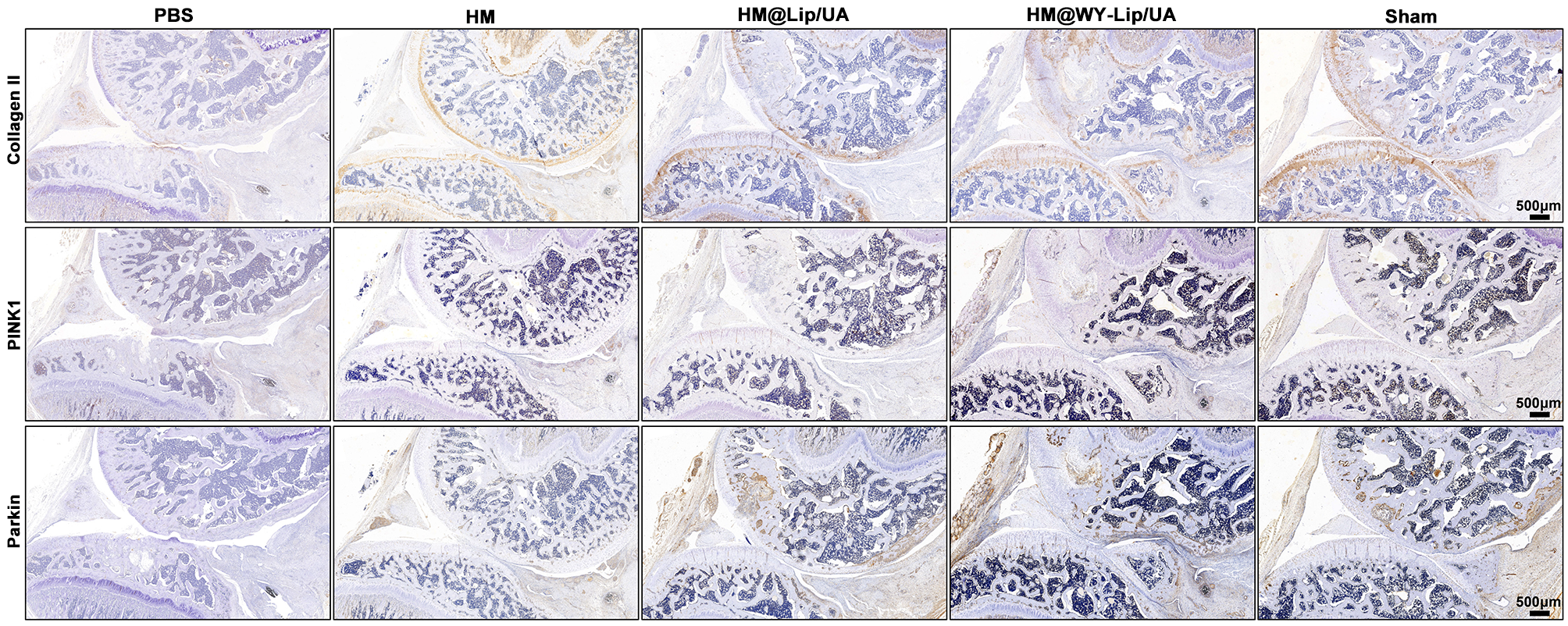


**Fig. S6** The panorama of slices of immunohistochemistry staining in Fig. 8. (A) Collogen II staining. (B) PINK1 staining. (C) Parkin staining.

**Table S1. Primers sequences used for qRT-PCR**

| **Gene** | **Forward primer (5'-3')** | **Reverse primer (5'-3')** |
| --- | --- | --- |
| *GAPDH*  *COL2A1*  *ACAN*  *MMP13*  *PINK1*  *Parkin* | ACGGCAAGTTCAACGGCACAG AATTTGGTGTGGACATAGGG AACTTCTTCGGAGTGGGTGGT  ACCCAGCCCTATCCCTTGAT GGAAAAGGCCCAGATGTCGT  GAACTGTGGCTGTGAGTGGA | CGACATACTCAGCACCAGCATCACAAGTATTTGGGTCCTTTGGG CAGGCTCTGAGACAGTGGGG  TCTCGGGATGGATGCTCGTA TGTTTGCTGAACCCAAGGCT  GGTGTTTCCCATGAGGTCGT |
